# Analysis of RAS and drug induced homo- and heterodimerization of RAF and KSR1 proteins in living cells using split Nanoluc luciferase

**DOI:** 10.1186/s12964-023-01146-9

**Published:** 2023-06-14

**Authors:** Lino Rohrer, Corinna Spohr, Carina Beha, Ricarda Griffin, Sandra Braun, Sebastian Halbach, Tilman Brummer

**Affiliations:** 1 Institute of Molecular Medicine and Cell Research (IMMZ), Zentrum für Biochemie und Molekulare Zellforschung (ZBMZ), Faculty of Medicine, University of Freiburg, Stefan-Meier-Str. 17, Freiburg, 79104 Germany; 2 German Cancer Consortium (DKTK), Partner Site Freiburg and German Cancer Research Center (DKFZ), Heidelberg, 69120 Germany; 3 Comprehensive Cancer Center Freiburg (CCCF), Medical Center, University of Freiburg, Faculty of Medicine, University of Freiburg, Freiburg, 79106 Germany; 4 Center for Biological Signalling Studies BIOSS, University of Freiburg, Freiburg, 79104 Germany

**Keywords:** BRAF, RAF1, KSR1, Sorafenib, Belvarafenib, NanoBit *Oplophorus* luciferase, LgBiT, SmBiT, KRAS, MAPK pathway

## Abstract

The dimerization of RAF kinases represents a key event in their activation cycle and in RAS/ERK pathway activation. Genetic, biochemical and structural approaches provided key insights into this process defining RAF signaling output and the clinical efficacy of RAF inhibitors (RAFi). However, methods reporting the dynamics of RAF dimerization in living cells and in real time are still in their infancy. Recently, split luciferase systems have been developed for the detection of protein–protein-interactions (PPIs), incl. proof-of-concept studies demonstrating the heterodimerization of the BRAF and RAF1 isoforms. Due to their small size, the Nanoluc luciferase moieties LgBiT and SmBiT, which reconstitute a light emitting holoenzyme upon fusion partner promoted interaction, appear as well-suited to study RAF dimerization. Here, we provide an extensive analysis of the suitability of the Nanoluc system to study the homo- and heterodimerization of BRAF, RAF1 and the related KSR1 pseudokinase. We show that KRAS^G12V^ promotes the homo- and heterodimerization of BRAF, while considerable KSR1 homo- and KSR1/BRAF heterodimerization already occurs in the absence of this active GTPase and requires a salt bridge between the CC-SAM domain of KSR1 and the BRAF-specific region. We demonstrate that loss-of-function mutations impairing key steps of the RAF activation cycle can be used as calibrators to gauge the dynamics of heterodimerization. This approach identified the RAS-binding domains and the C-terminal 14–3-3 binding motifs as particularly critical for the reconstitution of RAF mediated LgBiT/SmBiT reconstitution, while the dimer interface was less important for dimerization but essential for downstream signaling. We show for the first time that BRAF^V600E^, the most common BRAF oncoprotein whose dimerization status is controversially portrayed in the literature, forms homodimers in living cells more efficiently than its wildtype counterpart. Of note, Nanoluc activity reconstituted by BRAF^V600E^ homodimers is highly sensitive to the paradox-breaking RAFi PLX8394, indicating a dynamic and specific PPI. We report the effects of eleven ERK pathway inhibitors on RAF dimerization, incl. third-generation compounds that are less-defined in terms of their dimer promoting abilities. We identify Naporafenib as a potent and long-lasting dimerizer and show that the split Nanoluc approach discriminates between type I, I^1/2^ and II RAFi.

Video Abstract

**Supplementary Information:**

The online version contains supplementary material available at 10.1186/s12964-023-01146-9.

## Background

The RAS/RAF/MEK/ERK signaling pathway fulfils a critical role in almost all physiological and pathological conditions by controlling cell fate decisions, such as proliferation, survival differentiation status, migratory and invasive properties [1]. Consequently, all four core elements of this pathway are subject to alterations in cancer and are pursued pharmacologically, with several drugs already in clinical application or (pre)clinical development [2]. Spatio-temporal control of this pathway is achieved by dynamic protein–protein and protein-lipid interactions as well as a plethora of still incompletely defined post-translational modifications and feedback loops [3, 4]. Another layer of complexity is provided by the fact that mammalian genomes contain at least two paralogues for each core element of the RAS/RAF/MEK/ERK pathway. Although their gene products share overlapping functions, they possess distinct enzymatic activities and interactomes [5–7]. Moreover, the enzymes of all four tiers of the RAS/ERK pathway form homo- and heterodimers and thereby contribute to its fine-tuning by diversification of its signaling elements.

This notion has particularly developed from biochemical and genetic approaches that identified the RAF serine/threonine-kinases as critical signal integrators and pathway gatekeepers [3, 5, 8]. In mammals, the RAF family comprises the ARAF, BRAF and RAF1 (also known as CRAF) paralogues, which, despite the fact that they all act as MEK kinases, exhibit considerable differences in their tissue-specific expression levels, their regulation, enzymatic activities and their interactome [3, 9–12]. BRAF exhibits the highest MEK kinase activity followed by RAF1 and ARAF. Following the discovery of *BRAF* mutations 21 years ago, research into their pathomechanism and druggability has risen dramatically [8, 13].

An early branch-off in RAF evolution are the KSR proteins, which were originally considered as pseudokinases serving as a scaffold for the entire RAF/MEK/ERK module, but have more recently been recognized as allosteric activators of RAF proteins [7, 14, 15]. Nevertheless, their status as truly inactive pseudokinases, which was mostly established by the absence of the key catalytic lysine residue shared by most eukaryotic protein kinases, has been recurrently challenged [16, 17].

The concept that RAF activation requires dimerisation arose in 1996 when two groups demonstrated that RAF1 can be activated by fusion to either Gyrase B [18] or FKBP [19], two proteins undergoing dimerisation upon administration of coumermycin and FK506, respectively. In 2001, the Rapp laboratory demonstrated that active KRAS^G12V^ induced the formation of BRAF/RAF1 heterodimers without the need of artificial dimerizing domains [20]. In 2006, a seminal study observed for the first time a BRAF/RAF1 complex formed by endogenous proteins and demonstrated that this heterodimer possesses higher kinase activity than the respective homo- or monomers [21]. This finding suggested that cells might adjust RAF activity by controlling the formation of distinct homo- and heterodimers and hence the identification of the rules shaping the RAF dimer repertoire represents a future research task.

The relevance of dimerization for RAF activation is best understood from a structural perspective that was greatly enhanced by recent cryo-EM studies [22–25], and functional studies illuminating the activation cycle of RAFs [3, 8, 9, 26]. RAF isoforms share three conserved regions (CR) that encompass structurally defined subdomains [3]. The CR1 contains two subdomains involved in RAS binding, the RAS-binding domain (RBD) and the Cysteine-rich domain (CRD) [25]. The CR2 confers autoinhibition by recruiting 14–3-3 proteins, which stabilize the clamp formed by the N-terminal autoinhibitory moiety and the kinase domain. The CR3 starts with the N-terminal acidic (NtA) region, an area whose negative charges are critical for kinase activity [3, 27], and the kinase domain itself. Among other features, the kinase domain contains the activation segment (AS) and several residues involved in RAF dimerization, constituting the so-called dimer interface (DIF) [28]. A second 14–3-3 binding site around S621 (RAF1) or S729 (BRAF) is located at the C-terminal end of CR3 and loss of this evolutionary conserved motif drastically impairs the signaling output of RAF proteins [29–32]. It has been postulated for years and recently confirmed by cryo-EM that a 14–3-3 dimer serves as a match-maker by promoting dimerization of two BRAF protomers through binding to their C-terminal 14–3-3 binding sites [25].

But how does dimerisation activate RAF? First, there are two major autoinhibitory mechanisms that need to be overcome during RAF activation, one is the aforementioned 14–3-3 assisted clamping of the N- and C-terminal moieties and the other represents the destabilization of the inactive conformation of the kinase domain itself [8]. The latter is triggered by RAS dependent phosphorylation of the T^599^VKS^602^-motif in the AS [33] and represents a key event in the activation of wildtype BRAF (BRAF^WT^), but is dispensable in the context of the AS phosphorylation mimicking V600E mutation [34]. Negative charges in the AS, either introduced by phosphorylation or mutation, reorientate the so-called spine-residues within the kinase domain, which in turn exposes critical DIF residues such as R509, followed by enhanced dimerisation and complete activation [27, 34–37]. Histidine substitution of R509, or its equivalents in other RAF and KSR isoforms, impairs the formation of active RAF complexes, albeit the effects of this mutation vary between BRAF homo- or heterodimers [28, 29, 38]. In fact, the R509H mutation, despite its ability to block allosteric transactivation of one protomer by another in most if not all settings involving BRAF^WT^ and non-V600E mutants [29, 38], does not guarantee the conversion of RAF proteins into their monomeric form [39].

Mechanistic details on RAF dimerisation also contribute to our understanding of drug action and failure, as the binding of kinase inhibitors belonging to either type I, I^1/2^ or II, is heavily influenced by the dimerisation potential of RAF mutants. Moreover, these compounds themselves can promote or impair dimer formation [3, 8]. For example, RAF protomers bound to type I^1/2^ inhibitors like vemurafenib promote paradoxical transactivation of drug-free RAF protomers by a RAS- and DIF dependent allosteric transactivation mechanism, thereby contributing to secondary neoplasia and drug resistance [8, 40].

These findings spurred the interests into RAF dimerisation, although most methods used today, despite allowing deep insights even at the atomistic level, do not pay justice to the dynamics of RAF homo- or heterodimerization in living cells. So far, most studies either used co-immunoprecipitation experiments or proximity ligation assays, which assess the stability of dimers during the purification process from cell-free lysates or rely on fixed cells, respectively [21, 29, 41–44]. Moreover, the precipitating antibody might introduce a bias by favoring or discriminating against certain conformational states or post-translational modifications. An early approach to measure RAF dimerization in living cells was reported by Lavoie et al. (2013), who generated a bioluminescence resonance energy transfer (BRET)-based biosensor system to monitor the dimerization of isolated RAF kinase domains artificially tethered to membranes by a CAAX-box extension [45]. BRET, however, requires complex set-ups for detection. Split luciferase systems represent another approach that has been increasingly applied to the monitoring of protein–protein interactions, including RAF dimers and their modulation by kinase inhibitors [27, 39, 46, 47]. In such experiments, RAF proteins were fused to 416 and 152 amino acid long fusion partners derived from the fire fly (*Photinus*) luciferase, an enzyme of 65 kDa [47]. A very similar approach was applied using the click beetle (*Pyrearinus*) luciferase by generating moieties of 415 and 148 amino acids [44]. In contrast, the more recently established Nanoluc enzyme is based on a genetically modified luciferase (Nluc) from the deep-sea shrimp *Oplophorus gracilirostris* and can be split into considerably smaller 158 amino acid long LgBiT moiety (18 kDa) and an 11 amino acid SmBiT (1.3 kDa) peptide [48]. Upon interaction of their fusion partners, LgBiT and SmBiT weakly associate and reconstitute an active luciferase with high photon emission. As the LgBiT/SmBiT interaction is reversible, this so-called NanoBit system permits for the monitoring of temporal dynamics in living cells by addition of the cell permeable Nluc substrate Furimazine [49]. As Dixon et al. (2016) demonstrated in their technical report that the LgBiT/SmBiT complementation system can be in principle applied to BRAF/RAF1 heterodimers [48], we decided to explore this system in detail for its suitability to monitor the dimerisation dynamics of RAF and KSR proteins in various settings. In particular, we were interested in several aspects that were not addressed in the initial technical report by Dixon et al. (2016). Here, we report our experiences in using the Nluc approach to study RAF homo- and hetero-dimerization, either in response to physiological stimuli, oncogenic KRAS, kinase inhibitors and in the context of tumor-associated BRAF mutations.

## Materials and methods

### Plasmids

The pMIG expression vector and the cloning of pMIG/KRAS^G12V^ were described previously [50]. The pBiT1.1-N [TK/LgBiT], pBiT2.1-N [TK/SmBiT], pBiT1.1-C [TK/LgBiT] and pBiT2.1-C [TK/SmBiT] were purchased from Promega (Walldorf, Germany). Both pBiT1.1-N [TK/LgBiT] and pBiT2.1-N [TK/SmBiT] contain *bona fide* Kozak sequences and can be used for the expression of the Nluc moieties by themselves or for N-terminal fusions to the protein of interest. The pBiT1.1-C [TK/LgBiT] and pBiT2.1-C [TK/SmBiT] lack Kozak sequences and were used to clone the BRAF, RAF1 and KSR cDNAs in the multicloning site located 5’ of the open reading frames encoding the N-terminal G/S linker extended LgBiT and SmBiT proteins. First, the cDNAs for N-terminally HA-tagged BRAF and Myc-tagged RAF1 were amplified by PCR using Phusion-HF polymerase (New England Biolabs) and the pMIG/HA-BRAF and pMIBerry/Myc-RAF1 plasmids as templates, respectively [29]. The BRAF cDNA was amplified with the oligonucleotides *NheI/BmtI* Nterminal HA-tag (5’-ATAAGCTAGCATCGACCATGGCTTCTAGCTATCCTTATG-3’) and BRAF Cterm wo STOP *SalI* (5’-AAAGTCGACCCGTGGACAGGAAACGCACCATATCCC-3’). Likewise, the RAF1 cDNA was amplified using the oligonucleotides *NheI* Myc-tag (5’-AATATGCTAGCATAACCATGGCATCAGAGCAGAAGC-3’) and *XhoI* Raf1 rev wo STOP (5’-AAACTCGAGCCGAAGACAGGCAGCCTCGGGG-3’). Similarly, KSR1 was amplified from pMITom/Myc mKSR1 [29] using *NheIBmtI*Mycfwd (5- AAGCTAGCTCGACACCATGGCATCAGAGCAG-3’) and mKsr1woSTOP*SalI*rev (5’-AAGTCGACCCCATCTTTGGATTACCGGACTC-3’). This mKSR1 cDNA corresponds to the ENSEMBL canonical sequence, is widely used in the field [17, 51–54] and encodes for a full-length protein including critical structural elements such as the CC-SAM domain, whose genetic information is missing in commercially available human KSR1 cDNAs. As discussed below, the evolutionary conserved CC-SAM domain fulfils critical roles in the human orthologue as well [14, 51]. Not adjusting for variation arising from comparing alternative splice products in the databases, mKSR1 and hKSR1 are 87% identical at the protein level.

All forward primers introduce a Kozak sequence (accATGG) for optimal initiation of translation and all reverse primers remove the original STOP codons to allow the generation of an in-frame fusion with the LgBiT and SmBiT cDNAs. All amplicons were subcloned into pSCA-amp/kan (Stratagene) for propagation. The HA-BRAF cDNA was isolated from pSC-amp/kan by *BmtI*/*SalI* digestion and subcloned into *BmtI*/*XhoI* digested pBiT1.1-C [TK/LgBiT and pBiT2.1-C [TK/SmBiT]. The Myc-RAF1 cDNA was excised from pSC-amp/kan by *BamHI*/*XhoI* digestion and subcloned into *BglII*/*XhoI* digested pBiT1.1-C [TK/LgBiT and pBiT2.1-C [TK/SmBiT]. The Myc-KSR1 cDNA was isolated from pSC-amp/kan by *BmtI*/*SalI* digestion and subcloned into *Bmt*/*XhoI* digested pBiT1.1-C [TK/LgBiT and pBiT2.1-C [TK/SmBiT]. Site-directed mutagenesis was performed using *Pfu* polymerase and oligonucleotides described in Table S1. The fusion protein cassettes of all constructs were confirmed by sequencing.

### Cell culture

HEK293T cells were cultivated in DMEM medium (4.5 g/l glucose) supplemented with 10% fetal calf serum, 2 mM L-glutamine, 10 mM HEPES, 200 U/ml penicillin, 200 μg/ml streptomycin. Tissue culture media and additives were purchased from PAN Biotech (Aidenbach, Germany). *Mycoplasma* contamination was excluded by PCR using the service provided by Eurofins Genomics (Ebersberg, Germany). Kinase inhibitors were obtained from Selleck and dissolved in DMSO. Human recombinant EGF was purchased from Peprotech.

### Luciferase assays

One day before transfection, 2 × 10^6^ cells were plated in cell culture dishes (10 cm diameter; Sarstedt) with 10 ml cell culture medium. The next day, cells were transfected with the Polyethylenimine (PEI) method as described previously [24]. In brief, a 10 cm dish was transfected with 9 μg plasmid DNA (4 μg of a pBiT1.1 [TK/LgBiT] construct, 4 μg of a pBiT2.1 [TK/SmBiT] construct plus 1 μg of either pMIG empty vector or pMIG/KRAS^G12V^). To this end, 9 µg plasmid DNA were mixed with 1 ml DMEM (without any additives) containing 27 µl PEI solution (1 mg/ml), incubated at room temperature for 20 min and then added to the cells. Following incubation at 37 °C and 5% CO_2_ for 24 h, the transfected cells were harvested and adjusted to a cell density of 25,000 cells/100 µL cell culture medium. Then, 100 µl were transferred into wells of a black flat bottom 96-well plate and incubated overnight.

Prior to measurement, Nano-Glo® Live Cell Reagent (Promega) was generated by a 20-fold dilution of Nano-Glo® Live Cell Substrate in dilution buffer. Thereafter, 25 µl of Nano-Glo® Live Cell Reagent containing the Nluc substrate Furimazine was added to the cells. Long-term experiments were performed with the Vivazine and Endurazine substrates (Promega). Luminescence was either measured for 0.5 s per well using a Centro LB 960 luminometer (Berthold Technologies, Bad Wildbad, Germany) and the software MikroWin 2000 or on a Tecan Infinite M200 and associated software.

### Cell lysis, Western blotting and antibodies

Twenty-four hours after transfection, cells were harvested from 10 cm plates by trypsinization and seeded into black 96 well plates for Nluc assays. For the analysis of total cellular lysates (TCL), the remainder was lysed in 500 µl lysis buffer (50 mM Tris/HCl, pH 7.5; 1% Triton X-100; 137 mM sodium chloride; 1% glycerin; 1 mM sodium orthovanadate; 0.5 mM EDTA; 0.01 mg/ml leupeptin, 0.1 mg/ml aprotinin, 1 mM AEBSF) at 4 °C for 20 min. The lysate was cleared by centrifugation (15.800 × g, 4 °C) for 10 min, mixed with 5 × Laemmli buffer (150 mM Tris pH 6.8; 20% glycerol; 15% sodium dodecylsulfate; 15% β-mercaptoethanol 0.01% bromophenol blue), boiled at 98 °C for 5 min prior to storage at -20 °C and SDS-PAGE. Western blotting was performed using standard techniques. In brief, lysates were size-separated on a 10% SDS-PAGE gel followed by tank blot transfer to Polyscreen polyvinyl difluoride membranes (Perkin Elmer). Proteins of interest were detected with the following primary antibodies: anti-RAF1 (#9422), anti-MEK1/2 (#9122), Ras (G12V Mutant Specific) (D2H12; #14,412), anti-phospho-MEK1/2 (#9121), anti-ERK1/2 (#9102), anti-phospho-ERK1/2 (#9101), anti-MYC 9B11 (#2276), anti-HSP90 (#4874) (all from Cell Signaling Technology), anti-HA 3F10 (#11,867,431,001) (Roche), anti-B-Raf F7 (#sc-5284) and anti-pan-14–3–3 (#sc-1657) (all from Santa Cruz Biotechnology). Bound primary antibodies were detected using HRP-labelled secondary antibodies (Thermo Fisher) and a PeqlabTM Fusion Solo device and Fusion software (version 16.08).

### Co-immunoprecipitation

Two days after transfection, cells were lysed in 1 ml lysis buffer (50 mM Tris/HCl, pH 7.5; 0.5% NP-40; 137 mM sodium chloride; 1% glycerin; 1 mM sodium orthovanadate; 0.5 mM EDTA; 0.01 mg/ml leupeptin, 0.1 mg/ml aprotinin, 1 mM AEBSF) at 4 °C for 20 min. Following lysate clearance by centrifugation as described above, 100 µl lysate were taken for TCL analysis and the remainder (except the detergent insoluble pellet) was incubated with 0.4 µg anti-HA 3F10 or anti-Myc 9B11 antibodies for 1 h. Following addition of 40 µl Protein G-Sepharose bead slurry (4 Fast Flow, GE Healthcare), the antibody precipitates were captured on a rotating wheel at 4 °C for 3 h, washed six times with 1 ml lysis buffer and then resuspended in 60 µl lysis buffer, mixed with 5 × sample buffer and boiled at 98 °C for 5 min prior to storage at -20 °C and SDS-PAGE.

### Alignment analysis of KSR ortho- and paralogues

The alignment shown in Fig. 6A was constructed using the COBALT tool (https://www.ncbi.nlm.nih.gov/tools/cobalt/) and the following sequences (accession numbers) from placentals (*Mus musculus* (NP_038599.1), *Homo sapiens* (Q8IVT5.3)), Tasmanian devil (marsupial; *Sarcophilus harrisii*; XP_031823520.1), chicken (*Gallus gallus*; XP_425413.4), saltwater crocodile (*Crocodylus porosus*; XP_019401136.1), the frog *Xenopus tropicalis* (NP_001186688.1), zebrafish *Danio rerio* (XP_684771.5) and Elephant shark *Callorhinchus milii* (XP_007898884.1). *Drosophila* KSR isoform A (AAF52021.1) and murine KSR2 (NP_001108017.2) represent evolutionary more distant KSRs.

### Statistical analysis

Luciferase activities obtained from the MikroWin 2000 or Tecan Infinite M200 software were analyzed using GraphPad Prism Version 9. using the indicated statistical tests. If not stated otherwise, results were compared using one- or two-way ANOVA (Fisher’s LSD test). Data are presented as mean ± SEM and *p* values < 0.05 were considered statistically significant (* *P* < 0.05; ** *P* < 0.01; *** *P* < 0.001; **** *P* < 0.0001).

## Results

### Establishment of an Nluc complementation system to study RAF dimerization

**Fig. 1:**
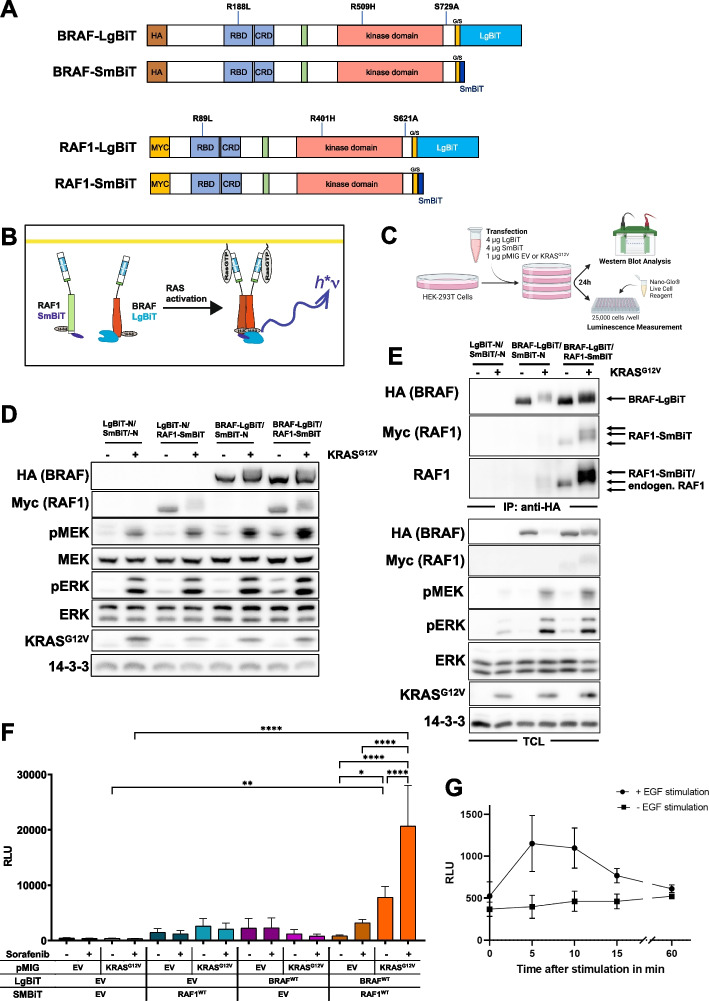
**A** Scheme of the RAF proteins used in this study. Proteins were epitope-tagged with either HA- or Myc-epitopes (not drawn to scale). At their C-termini, BRAF and RAF1 were linked to either the 158 amino acid (aa) long LgBiT or the 11 aa long SmBiT by a 15 aa glycine/serine-rich (G/S) linker (not drawn to scale). **B** Assay principle. Each protomer is either fused to the LgBiT or SmBiT moiety of Nluc. If the two RAF protomers dimerize, the Nluc moieties will assemble and emit photons (h*ν) in the presence of Furimazine. Shown is a set of two protomers in either an inactive (green) or active (red) conformation. Upon dimerization, the inactive protomer becomes allosterically activated. **C** Assay workflow created with BioRender.com. **D** Western blot of total cell lysates (TCL) from HEK293T cells transfected with the indicated LgBiT/SmBiT pairs, either with pMIG control vector (-) or pMIG KRAS^G12V^ ( +). Detection of 14–3-3 proteins serves as loading control. **E** HA-BRAF-LgBiT was immuno-purified using anti-HA antibodies and co-immunoprecipitated RAF1 was detected with anti-Myc or anti-RAF1 antibodies. TCL analysis confirms RAS/RAF/MEK/ERK-pathway activation by KRAS^G12V^. **F** Relative luminescence (RLU) of HEK293T cells transfected with the indicated LgBiT/SmBiT pairs and with either pMIG (EV) or pMIG KRAS^G12V^. Cells were either treated with 10 µM Sorafenib or DMSO (vehicle) for 4 h prior to measurement. Shown is the mean of three biological replicates. Statistical significance was assessed using a mixed effects analysis with an uncorrected Fisher’s LSD test (single pooled variance). **G** HEK293T cells expressing BRAF-LgBiT and RAF1-SmBiT were either left untreated or stimulated with 100 ng/ml EGF. Shown is the mean of three technical replicates with error bars indicating standard deviation

Based on a previous report showing that the larger *Pyrearinus* split luciferase fragments are best tolerated at the C-terminus of BRAF and RAF1 [44], we generated expression vectors allowing the production of BRAF and RAF1 with LgBiT or SmBiT moieties fused to their C-termini (Fig. 1A). To distinguish these fusion proteins from their endogenous counterparts, in particular as the SmBiT only extends the RAF proteins by 11 amino acids, we added hemagglutinin (HA) or Myc epitope tags to their N-termini. Both N-terminal extensions are widely used in RAF research and no interference with RAF activation or downstream signaling has been reported so far [29, 43, 55, 56].

To test the suitability of the Nluc system for studying RAF homo- and heterodimerization and to confirm the biological activity of these tagged RAF proteins, we transiently transfected HEK293T cells with constructs allowing the analysis of BRAF/RAF1 heterodimerization and the appropriate LgBiT-N/SmBiT-N control vectors encoding either LgBiT or SmBiT proper. The co-expression of LgBiT/SmBiT proper allows to determine Nluc background activity as these proteins are not supposed to interact regularly. In contrast, if LgBiT/SmBiT association is promoted by their fusion partners, i.e. RAF1 and BRAF, a functional Nluc will be reconstituted and emit photons in the presence of its substrate (Fig. 1B).

In most of the following experiments, HEK293T cells transfected with various RAF-LgBiT and RAF-SmBiT expression vectors were split into a subpopulation for Western blot analyses and luminescence measurements (Fig. 1C). First, we confirmed that RAF proteins could be efficiently expressed and remain functional. To this end, we included the bi-cistronic expression vector pMIG/KRAS^G12V^ (or empty pMIG as control) to induce RAF/MEK/ERK pathway activation. As shown in Fig. 1D, the Myc-tagged RAF1-SmBiT and HA-tagged BRAF-LgBiT could be efficiently expressed. KRAS^G12V^ induced MEK/ERK phosphorylation and the characteristic phosphorylation mediated electrophoretic mobility shifts well-documented for BRAF and RAF1 [29, 57]. Basal MEK/ERK phosphorylation was highest in cells co-expressing BRAF-LgBiT and RAF1-SmBiT compared to cells ectopically expressing only one isoform or co-expressing the Nluc moieties only. Transfection with expression vectors for LgBiT and SmBiT proper yielded the lowest level of MEK/ERK phosphorylation, indicating that the increased pMEK/pERK levels in cells transfected with Myc-tagged RAF1-SmBiT and HA-tagged BRAF-LgBiT were specifically triggered by these fusion proteins (Fig. 1D/E). We then confirmed in co-immunoprecipitation experiments that the BRAF-LgBiT was not only able to interact with RAF1-SmBiT but also with endogenous RAF1, in particular if dimerization was promoted by KRAS^G12V^ (Fig. 1E). Taken together, Myc-tagged RAF1-SmBiT and HA-tagged BRAF-LgBiT fusion proteins respond to signals provided by oncogenic KRAS^G12V^ and retain their ability to dimerize and to trigger ERK pathway activation.

Next, we assessed the degree of Nluc reconstitution by assaying luciferase activity. Cells transfected with the RAF-less LgBiT-N/ SmBiT-N expression vector pair displayed very little luciferase activity that was only enhanced by less than two-fold upon co-transfection of BRAF-LgBiT and RAF1-SmBiT expression vectors along with empty pMIG (Fig. 1F). Commensurate with the co-immunoprecipitation data in Fig. 1E, co-expression of both BRAF-LgBiT and RAF1-SmBiT with KRAS^G12V^ increased luminescence by ninefold, indicating a significant increase in heterodimer formation. Administration of the dimer promoting RAFi Sorafenib further increased Nluc activity in HEK293T cells co-expressing BRAF-LgBiT and RAF1-SmBiT, in particular in cooperation with KRAS^G12V^. This effect was not observed in the negative controls transfected with the RAF-less LgBiT-N/ SmBiT-N expression vector pair. This data fits well to previous co-immunoprecipitation experiments demonstrating that active RAS increases BRAF/RAF1 heterodimers, which are further stabilized by Sorafenib [29, 34, 43]. Moreover, a co-immunoprecipitation experiment confirmed that the LgBiT and SmBiT extensions on BRAF and RAF1 do not counteract the strong dimerization promoting effect of Sorafenib (Figure S1**)**.

Interestingly, compared to cells expressing LgBiT-N/SmBiT-N (baseline luminescence), cells transfected with either the BRAF-LgBiT/SmBiT-N or LgBiT-N/SmBiT-RAF1 pairs displayed a trend for enhanced Nluc activity (Fig. 1F). One potential explanation for this phenomenon could be that LgBiT or SmBiT are better expressed or stabilized, if their N-termini are extended by a polypeptide. As a result, more LgBiT and SmBiT moieties might become available for spontaneous Nluc reconstitution and hence background activity will increase. However, neither expression of KRAS^G12V^ nor sorafenib significantly elevated this Nluc background activity (Fig. 1F).

We also asked whether Nluc complementation could be induced by growth factors. Therefore, HEK293T cells co-expressing BRAF-LgBiT/RAF1-SmBiT were stimulated with EGF over a time course of 60 min. Commensurate with the typical EGF induced ERK activation and BRAF/RAF1 heterodimerization kinetics [56], Nluc activity was highest at 5 min and then gradually declined over the next 55 min (Fig. 1G). We also conducted a side-by-side comparison of the BRAF/RAF1 heterodimer driven Nluc reconstitution induced by either EGF alone, KRAS^G12V^ or the combination of both (Figure S2). These analyses showed that KRAS^G12V^-stimulated dimerization represents a much stronger stimulus than EGF stimulation alone (Figure S2). Taken together, our data show that Nluc activity exhibited by cells expressing a BRAF-LgBiT/RAF1-SmBiT pair can be strongly and specifically increased by conditions promoting RAF heterodimerization and that the association of these fusion proteins is dynamic and reversible.

### Gauging the Nluc reporter system with mutants impeding RAF dimerization

As outlined above, RAF dimerization represents a complex process involving three key events: i.) membrane recruitment by active RAS and displacement of the autoinhibitory N-terminal moiety followed by exposure of the kinase domains, ii.) “matchmaking” by a 14–3-3 dimer binding to the C-terminal 14–3-3 binding sites in the RAF protomers and iii.) formation of stable dimers promoting allosteric transactivation via the DIF [8, 22]. However, which of these mechanisms represents the critical one for RAF dimerization in living cells remains unresolved. Therefore and because structural RAF mutants have not been investigated in detail with split luciferase reporters, we studied the contribution of these three events for BRAF/RAF1 heterodimerization by using BRAF in the LgBiT and RAF1 in the SmBiT context.

**Fig. 2:**
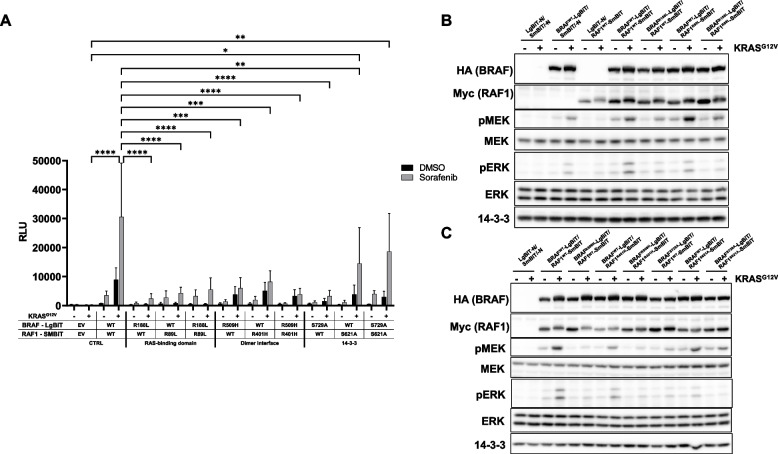
Effects of mutations suggested to impair RAF dimerization. **A** HEK293T cells were transfected with the indicated LgBiT/SmBiT pairs, either with empty pMIG control vector (-) or pMIG KRAS^G12V^ ( +). Cells were either treated with 10 µM Sorafenib or DMSO (vehicle control) for 4 h prior to measurement. Shown is the mean of three biological replicates. (**B**) and (**C**) Western blot analysis of TCLs from cells transfected with the indicated LgBiT/SmBiT pairs, either with empty pMIG control vector (-) or pMIG KRAS^G12V^ ( +). Shown is a representative experiment from two biological replicates

First, we investigated the role of the RAS/RAF interaction by introducing the well-known R89L and R188L substitutions [58, 59] into the RBDs of RAF1 and BRAF, respectively. As shown in Fig. 2A, RBD mutations in both BRAF and RAF1 impaired RAF dimerization, even if only one dimerization partner carried an RBD mutation. Basal Nluc activity of cells co-expressing RBD mutants and co-transfected with the empty pMIG control vector was barely distinguishable from that of negative control expressing only LgBiT and SmBiT proper. Remarkably, cells expressing BRAF^R188L^ and/or RAF^R89L^ were severely impaired in inducing Nluc activity in response to KRAS^G12V^. Our results tie in with the original and recently cryo-EM corroborated concept that RAS mediated relief from the auto-inhibited conformation represents a prerequisite for dimerization [20, 25]. Heterodimerization promoted by the combination of KRAS^G12V^ and Sorafenib was strongly impaired, even if only one Nluc complementation partner carried an RBD mutation. Nevertheless, Sorafenib was still able to slightly increase Nluc activity in cells expressing RBD mutants of BRAF and RAF1, suggesting that drug induced heterodimerization is not entirely RAS dependent.

Although the DIF plays an essential role for allosteric transactivation and a critical role for the formation of BRAF homo- and to a lesser extent BRAF/RAF1 heterodimers that are stable enough to survive co-immunoprecipitation procedures [29, 38], little is known as to how DIF mutations impair RAF dimerization in living cells. Therefore, we asked how DIF mutations in BRAF (R509H) and RAF1 (R401H) would impact on heterodimerization (Fig. 2A). Interestingly, we did not observe a significant reduction in Nluc reconstitution in cells in which dimerization is induced by KRAS^G12V^ only. At first glance, this data might surprise, but agrees with our earlier co-immunoprecipitation studies showing that DIF mutations do not completely abolish BRAF/RAF1 heterodimerization, despite having a severe effect on transactivation and downstream signaling [29]. In contrast, introduction of a DIF mutation in only one of the protomers significantly impaired dimerization induced by the combination of KRAS^G12V^ with Sorafenib, which was further reduced upon introduction of DIF mutations into both protomers.

Although R509 plays a very critical role in allosteric transactivation, its histidine substitution is often insufficient to abrogate BRAF dimerization in co-immunoprecipitation experiments, in particular in the context of BRAF/RAF1 heterodimers [29]. This is probably best explained by experimental conditions such as lysis buffer stringency and the fact that the DIF is composed by multiple and non-contiguous amino acid residues that could compensate for the loss of R509 [28, 60]. We thus asked how the introduction of additional DIF mutations, which are known to further reduce dimerization propensity, would affect BRAF-LgBiT/RAF1-SmBiT heterodimerization (Fig. S3). To this end, we tested the 3 × mutation of which we had shown previously that it further reduces the stability of BRAF/RAF1 heterodimers in co-immunoprecipitation experiments [29]. This mutation introduces the R509H/L515G/M517W substitutions into the R^509^HxNΦΦL^515^FM^517^G motif at the C-terminal half of the central DIF with x and Φ denoting any or a hydrophobic amino acid residue, respectively.

Based on data by Hu et al. (2013), who showed for the RAF1 kinase domain that the combination of the R401H mutation (R509H equivalent) with the H369A substitution further reduced the association of a wildtype RAF1 kinase domain to RAF1^R401H^ kinase domain in a split click beetle luciferase reconstitution system [27], we replaced this evolutionary conserved histidine [28], H477 in BRAF, by an alanine residue in the BRAF^R509H^-LgBiT background. In Nluc reconstitution assays, we observed no significant differences in the association between BRAF^R509H^-LgBiT, BRAF^3x^-LgBiT and BRAF^H477A/R509H^-LgBiT with RAF1-SmBiT, albeit there was a trend for less heterodimers formed between wildtype RAF1 and BRAF mutants with composite DIF mutations in Sorafenib treated cells expressing KRAS^G12V^ (Fig. S3A). In co-immunoprecipitation experiments, however, we could reproduce with the LgBiT/SmBiT fusion proteins our original findings [29], showing that the 3 × mutation more severely impacts on RAS and/or Sorafenib induced BRAF/RAF1 heterodimerization than R509H alone (Fig. S3B). The BRAF^H477A/R509H^-LgBiT double mutant was also co-purified to a lesser extent with RAF1 in the co-immunoprecipitation experiment. These data suggest the Nluc reconstitution system is more sensitive to detect low-level but, due to the lack of transactivation capacity, physiologically less relevant heterodimerization between BRAF and RAF1 than the co-immunoprecipitation experiments that only reveal interactions stable enough to persist post-lysis conditions. One also has to keep in mind that we, in contrast to the aforementioned experiment by Hu et al. (2013), were using full-length RAF proteins instead of isolated kinase domains.

Next, we investigated the relevance of the C-terminal 14–3-3 binding site for dimerization by introducing the S621A and S729A mutations into RAF1 and BRAF, respectively. As shown in Fig. 2A, introduction of a 14–3-3 binding site mutation into either BRAF or RAF1 provoked a trend for reduced KRAS^G12V^ driven heterodimerization, indicating that both protomers require their C-terminal 14–3-3 binding site for this process. Interestingly, Sorafenib partially overcame this defect suggesting that the drug induced conformational changes discussed below might compensate for the lack of 14–3-3 assisted dimerization.

To confirm expression of the mutant BRAF-LgBiT and RAF1-SmBiT fusion proteins and to monitor their impact on basal and KRAS^G12V^ triggered MEK/ERK phosphorylation, we performed Western blot analyses (Fig. 2B/C and Fig. S3B). Interestingly, while RBD mutations in only one protomer already had a severe effect on Nluc complementation differentials (Fig. 2A), KRAS^G12V^ induced MEK/ERK phosphorylation was reduced but still clearly detectable in cells expressing RBD mutations of BRAF and RAF1, either singly or in combination (Fig. 2B). Thus, dimerization behavior as a read-out by protomer-assisted Nluc reconstitution did not strictly correlate with downstream MEK/ERK phosphorylation, although it should be kept in mind that MEK/ERK phosphorylation could be also mediated by the endogenous RAF isoforms present in HEK293T cells.

As expected from previous studies using full-length RAF proteins [29, 56], DIF mutations in BRAF and, albeit to a lesser extent in RAF1, impaired MEK/ERK phosphorylation when co-expressed with their partner protomer in its wildtype context (Fig. 2C and Fig. S3B). Co-expression of BRAF^R509H^-LgBiT and RAF1^R401H^-SmBiT further reduced MEK/ERK phosphorylation, indicating that these proteins require an intact DIF for downstream signaling. Likewise, introduction of the S729A and S621A mutations in BRAF-LgBiT and RAF1-SmBiT impaired downstream signaling, in particular if this defect was introduced into both protomers. This indicates that 14–3-3 recruitment to the C-termini of these fusion proteins is as critical for their biological activity as it has been established for their unmodified counterparts [29–32]. In essence, the LgBiT and SmBiT extensions do no not alter the MEK/ERK phosphorylation potential established for RBD, DIF and C-terminal 14–3-3 binding motif mutants.

### The Nluc reporter system is suitable to monitor drug modulated RAF dimerization

RAF inhibitors (RAFi) exert contrasting effects on the orientation of the αC-helix and the DFG motif of the activation segment [40, 61]. Of note, the orientation of R506 in the αC-helix of BRAF influences the exposure of the critical DIF residue R509 and thus influences inhibitor-induced RAF dimerization [61]. Based on their effects on the relative orientation of the αC-helix and the DFG motif, RAFi can be categorized into three groups. Type I inhibitors lock the kinase domains of RAF proteins in their active “αC-helix-in/DFG-in/R506in” conformation, while type II compounds, e.g. the first-generation inhibitor Sorafenib, stabilize them in their inactive “αC-helix-in/DFG-out/R506in” conformation and hence are supposed to stabilize RAF dimers, as it has been demonstrated for Sorafenib in co-immunoprecipitation experiments [29, 43]. The BRAF^V600E^ selective inhibitors Vemurafenib, Dabrafenib, and, at least according to predictions, also Encorafenib represent so-called I^1/2^ inhibitors [40]. These drugs induce an “αC-helix-out/DFG-in/R506in” conformation, which not only promotes dimerization, but also prevents inhibitor uptake of the drug-free protomer partner by negative allostery [61, 62]. The recently developed paradox breakers, e.g. PLX8394, are based on vemurafenib and dabrafenib, but induce an αC-helix-in/DFG-in/R506out” conformation and thereby avoid the effects of the original type I^1/2^ inhibitors [61, 62].

**Fig. 3:**
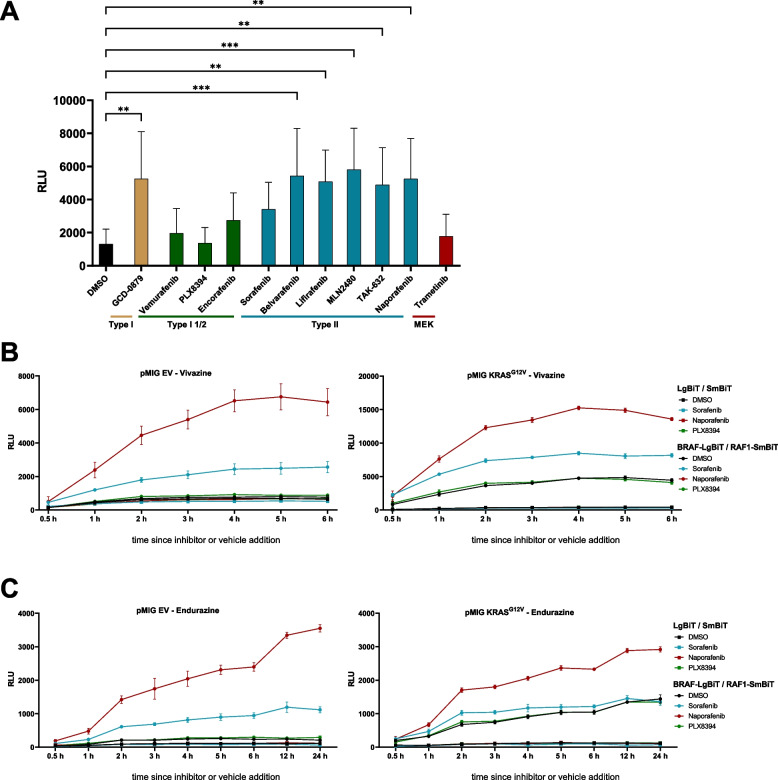
The Nluc system reports RAF inhibitor induced dimerization dynamics. **A** Effects of ten different RAF inhibitors and the MEK Inhibitor trametinib on BRAF-LgBiT/RAF1-SmBiT heterodimerization. HEK293T cells were transfected with expression vectors for BRAF-LgBiT, RAF1-SmBiT and pMIG. Cells were treated with the following inhibitor concentration 4 h prior to measurement: GDC-0879 (1 µM), Vemurafenib and PLX8394 (both 1 µM), Encorafenib (0.5 µM), Sorafenib (10 µM), Belvarafenib (1 µM), Lifirafenib (1 µM), MLN2480 (3 µM), TAK-832 and Naporafenib (both 1 µM) and Trametinib 0.05 µM). Classification of inhibitors is based on Ref. [40]. DMSO serves as vehicle control. The diagram shows the mean of three biological replicates, each carried out in three to five technical replicates. **B** and **C** Long-term measurement of BRAF/RAF1 heterodimerization using the Nluc substrates Vivazine (**B**) and Endurazine (**C**). HEK293T cells were transfected with expression vectors for BRAF-LgBiT and RAF1-SmBiT, either in combination with pMIG e.V. (left) or pMIG KRAS^G12V^ (right). Cells were treated with the indicated inhibitor concentrations and luminescence was measured at the indicated time points. DMSO serves as vehicle control. The diagram shows the mean of five technical replicates. Note that the y-axis scales differ in the Vivazine experiment between empty pMIG control vector (EV) and pMIG KRAS^G12V^ transfected cells, due to the higher Nluc activity displayed by the latter

While several type I^1/2^ and II inhibitors are in clinical use or advanced preclinical development, type I compounds have not progressed to these stages. The third generation RAFi comprise the aforementioned “paradox breakers” and several promising type II compounds. The latter appear particular promising for targeting dimeric RAF, as they are able to inhibit both protomers within a preformed dimer [40, 61]. They have been also termed as pan-RAFi, although more recent data indicate that Naporafenib (also known as LXH254), Belvarafenib and MLN2480 (also known as Tovorafenib or TAK-580) spare ARAF [63–65]. Despite these insights, many questions remain and the effects of several third generation inhibitors on the conformation and dimerization behavior of RAFs remain ill-defined [40]. Therefore, and to evaluate the suitability of the Nluc complementation system for inhibitor characterization, ten RAF inhibitors and one MEK inhibitor were analyzed concerning their influence on BRAF-RAF1 heterodimerization (Fig. 3A). Inhibitor concentrations were chosen based on a literature survey or our previous experiences and were aimed, if information was available, to pharmacological meaningful concentrations [29, 38, 63, 64, 66–69]. As shown in Fig. 3A, the type I compound GDC-0879 as well as all type II inhibitors promoted BRAF/RAF1 dimerization as reflected by increased luminescence. As expected from the aforementioned studies on type I^1/2^ inhibitors, Vemurafenib did not promote a significant increase in luminescence. This can be explained by the fact that dimers promoted by this type I^1/2^ inhibitor are less stable than those formed by other drugs, in particular type II compounds, due to the special effects of vemurafenib on the orientation of the αC-helix of BRAF [3, 62]. Our mean differential in BRAF/RAF1 heterodimerization of merely 1.5-fold between DMSO and vemurafenib treated cells also agrees with that of a previous publication using a split click beetle luciferase reporter [44]. PLX8394, which was developed from vemurafenib and in which dimer promoting features are absent [70], failed to induce BRAF/RAF1 heterodimers (Fig. 3A). The MEK inhibitor trametinib failed to enhance Nluc activity, indicating that loss of ERK signaling, which could have stabilized dimers by loss of negative feedback [21, 71], cannot explain the RAF inhibitor induced alterations in the BRAF-LgBiT/RAF1-SmBiT complementation rate.

Apart from identifying inhibitor effects on dimerization at a specific time point, the NanoBiT system could also serve as a platform for kinetic experiments, e.g. to identify the appropriate incubation period without the need to conduct time consuming co-immunoprecipitation experiments. Thus, we asked whether the 4 h treatment period used in Fig. 3A, which was based on previous publications [29, 43], was indeed appropriate, or whether shorter or longer incubation periods as reported for other co-immunoprecipitation experiments [63, 64, 68], would be more meaningful. Therefore, we used the Furimazine esters Vivazine and Endurazine for long-term measurements as our initial experiments confirmed the manufacturer’s statement that Nluc signals, depending on the initial activity, already decay 30 to 60 min after Furimazine addition (data not shown). Vivazine and Endurazine are more stable than Furimazine, which rapidly becomes de-esterified by cellular esterases, and thereby provide a more long-term supply of the Nluc substrate (public information provided by the manufacturer on their website). Due to their different de-esterification rates, however, these substrates differ in their photon emission. As shown in Fig. 3B, a maximum or plateau in BRAF-LgBiT/RAF1SmBiT heterodimers induced by the type II inhibitors Sorafenib and Naporafenib was also observed at the 4 h time point initially chosen for the experiments in Fig. 3A. At this time point, the combination of KRAS^G12V^ and Naporafenib induced an approximately 20-fold increase in Nluc activity compared to DMSO treated, empty control vector transfected HEK293T cells expressing BRAF-LgBiT and RAF1-SmBiT. This data demonstrates that KRAS^G12V^ induced BRAF/RAF1 heterodimers are potently stabilized by this dimer targeting RAFi.

Using Endurazine, a substrate that, according to the manufacturer, provides an initially weaker but more long-lasting Nluc signal than Furimazine and Vivazine, we were able to show that Naporafenib continues to drive BRAF-LgBiT/RAF1SmBiT dimers for up to 24 h (Fig. 3C). This longevity of Naporafenib induced RAF heterodimers agrees with co-immunoprecipitation experiments [64].

### Studying RAF homo- vs. heterodimerization with the Nluc reporter

**Fig. 4:**
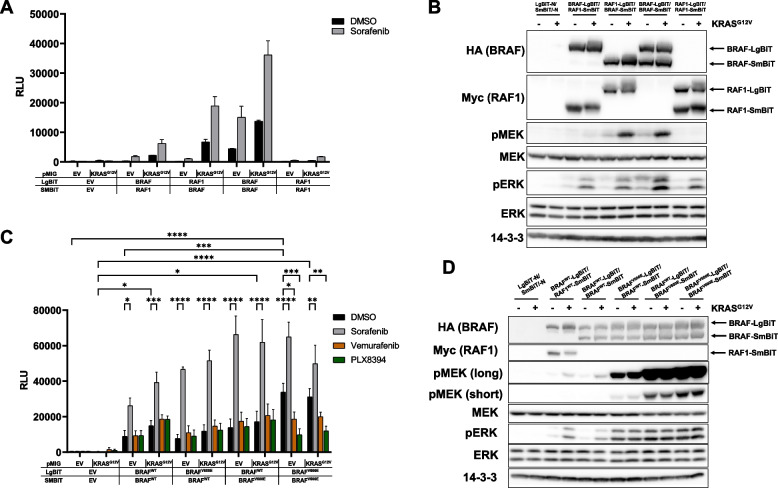
The Nluc system can be used to measure association of BRAF and RAF1 homo- and heterodimers and reveals an increased homodimerization potential of BRAF^V600E^ in living cells. **A** HEK293T cells were transfected with the indicated plasmid combinations. Shown is the mean of three technical replicates. **B** Western blot analysis of cells from the experiment shown in (**A**) using the indicated antibodies. Shown is a representative experiment from two biological replicates. **C** HEK293T cells were transfected with the indicated plasmid combinations. Shown is the mean of three biological replicates. **D** Western blot analysis of cells from a single experiment described in (**C**) using the indicated antibodies. Shown is a representative experiment from three biological replicates

Next, we asked whether BRAF/BRAF and RAF1/RAF1 homodimers can be also monitored with the Nluc complementation approach and applied the aforementioned expression vectors encoding BRAF-SmBiT and RAF1-LgBiT (Fig. 1A). As shown in Fig. 4A, the BRAF-LgBiT/BRAF-SmBiT pair produced prominent Nluc activity, which was further enhanced by KRAS^G12V^ and/or Sorafenib. Interestingly, the RAF1-LgBiT/RAF1-SmBiT homo-dimer produced far less Nluc activity than the two BRAF/RAF1 combinations in response to KRAS^G12V^, which might reflect the recently reported striking preference of mutant KRAS towards the RBD of BRAF [72]. While our study was in preparation, Murphy et al. showed with the Nluc system that murine embryonic fibroblasts (MEFs) expressing oncogenic NRAS^Q61R^ also displayed a lower fold change for RAF1-LgBiT/RAF1-SmBiT homodimers than for BRAF-LgBiT/BRAF-SmBiT homo- and BRAF-LgBiT/RAF1-SmBiT heterodimers [73]. Thus, BRAF appears to possess a higher intrinsic dimerization propensity than RAF1.

The Western blot analysis in Fig. 4B demonstrates that all fusion proteins were efficiently produced and that cells co-expressing RAF1-LgBiT/BRAF-SmBiT and BRAF-LgBiT/BRAF-SmBiT dimer displayed enhanced MEK phosphorylation compared to the other combinations.

Although the considerably weaker MEK/ERK phosphorylation displayed by cells expressing RAF1-LgBiT/RAF1-SmBiT homodimers fits well to the data in Fig. 4A and the notion that these homo-dimers are less active than BRAF/RAF1 heterodimers and BRAF/BRAF homodimers [21], it is impossible to directly compare the Nluc activities of BRAF-LgBiT/BRAF-SmBiT and RAF1-LgBiT/RAF1-SmBiT homodimers, as the distinct epitope-tags cannot be used to confirm equal expression between HA- and Myc-tagged RAFs. Unfortunately, pan-RAF antibodies that were generated in the late 1980s [74, 75] and could have addressed this question were not available. Nevertheless, our experimental set-up clearly reveals that BRAF, no matter whether it was expressed in the LgBiT or SmBiT context, enhances the Nluc complementation potential of both RAF1-LgBiT and -SmBiT.

The most common BRAF oncoprotein, BRAF^V600E^, neither requires an intact DIF nor 14–3-3 binding for MEK activation and transformation [29, 42, 76]. Moreover, BRAF^V600E^ selective drugs like Vemurafenib exhibit negative allostery and hence only bind their target in its monomeric state. These findings suggested that BRAF^V600E^ exists and signals as a monomer in living cells [13, 77]. Several independent groups, however, have confirmed now that BRAF^V600E^ forms particularly stable homodimers in co-immunoprecipitation experiments and is incorporated in multi-protein complexes whose size correlates with an intact DIF [29, 36, 78]. More recently, Yuan et al.demonstrated that BRAF^V600E^ phosphorylates MEK in a dimer-dependent manner [39]. All these data invite for an *in cellulo* analysis of the dimerization behavior of BRAF^V600E^. Indeed, Fig. 4C confirms that BRAF^V600E^-LgBiT/BRAF^V600E^-SmBiT dimers are formed almost four-fold more efficiently as in their wildtype context, while introduction of the V600E mutation into a single protomer only mildly increased luminescence. Interestingly, this increased self-association of BRAF^V600E^ was not further promoted by KRAS^G12V^, supporting the notion that this dimerization behavior reflects the special conformation of the kinase domain of this oncoprotein, which is induced by the mutation-specific salt-bridge established by E^600^ [36]. In agreement with structural studies showing that the BRAF^V600E^ selective inhibitor Vemurafenib rather induces instable dimers [3] and that PLX8394 acts as a “paradox breaker” [79], these drugs suppressed the prominent Nluc activity of BRAF^V600E^-LgBiT/BRAF^V600E^-SmBiT co-expressing cells (Fig. 4C). To the best of our knowledge, our Nluc approach documents for the first time the formation of stable but drug-sensitive homodimers of full-length BRAF^V600E^ in living cells. As shown in Fig. 4D, all BRAF-LgBiT and BRAF-SmBiT proteins were expressed to similar levels and the V600E substitution induced high levels of MEK/ERK phosphorylation. Interestingly, the signaling potential of BRAF^V600E^ was more pronounced in the SmBiT than in the LgBiT context.

### New insights into RAF/KSR dimerization by Nluc technology

**Fig. 5:**
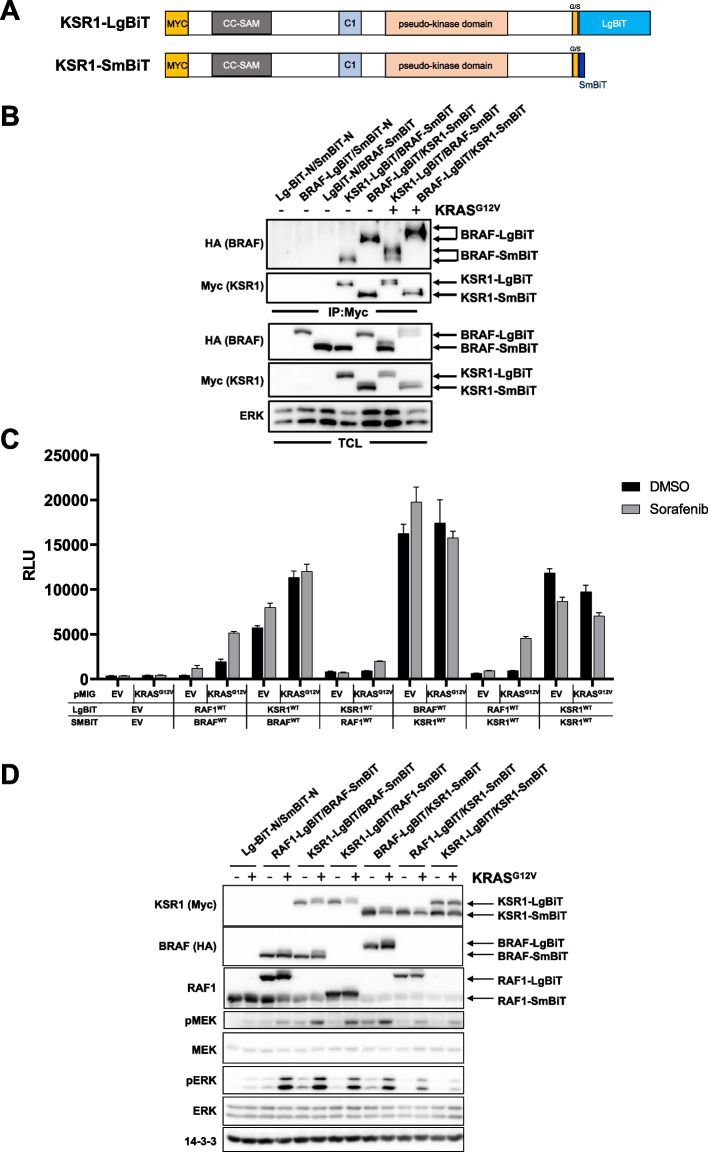
The Nluc system reveals homodimerization of KSR1 and its interaction with BRAF. **A** Scheme of the KSR1 fusion proteins used in this study. Proteins were epitope-tagged at their N-termini with Myc-tags (not drawn to scale). At their C-termini, KSR1 proteins are linked to either the 158 amino acid (aa) long LgBiT or the 11 aa long SmBiT via a 15 amino acid glycine/serine -rich (G/S) linker (also not drawn to scale). Please refer to Ref. [14] and the main text for details on protein domains. **B** Expression vectors for the indicated Myc-tagged KSR1 and HA-tagged BRAF proteins were co-transfected into HEK293T cells with either empty pMIG (-) or pMIG/KRAS.^G12V^ ( +). KSR1-LgBiT and -SmBiT fusion proteins were immunoprecipitated using anti-Myc antibody. Following Western blotting, immunoprecipitates (IP) and TCLs were probed with anti-HA and anti-Myc antibodies to detect the specific interaction between KSR and BRAF and to confirm successful expression. Note that HA-BRAF is not co-precipitated by anti-Myc antibodies, if Myc-KSR1 is not co-expressed, indicating that HA signals in anti-Myc immunoprecipitates reflect specific interactions. **C** HEK293T cells were transfected with the indicated plasmid combinations and treated with Sorafenib or DMSO for 4 h. Shown is the mean of three technical replicates. **D** Western blot analysis of cells from the experiment shown in (**B**) using the indicated antibodies. Shown are representative results from two biological replicates, except for the BRAF-LgBiT/KSR1-SmBiT pair (*n* = 1)

KSR1 was originally considered to act as a pure scaffold protein organizing the three kinases of the ERK axis in a similar way, as it was described for Ste5 or Pbs2 in yeast MAPK pathways (reviewed in [80]). More recent studies show that, as a result of their similarities to RAF kinase domains, the KSR1 pseudokinase domain also serves as an important allosteric activator of BRAF [14, 28, 81]. Moreover, KSR1 expression levels positively correlate with an elevated ERK pathway ground state [82]. Nevertheless, as the spectrum of RAF/KSR heterodimers remains ill-defined, we asked whether the dimerization behavior of this pseudokinase can be monitored by Nluc methodology as well. Therefore, we generated KSR1 constructs with C-terminal LgBiT or SmBiT extensions (Fig. 5A) and confirmed their expression in HEK293T cells (Fig. 5B). As little is known about the role of the KSR1 C-terminus in RAF dimerization, we first tested whether KSR1, either in its LgBiT or SmBiT context, is still able to interact with BRAF-LgBiT or -SmBiT. As shown in Fig. 5B, Myc-tagged KSR1-LgBiT and -SmBiT proteins efficiently purified HA-tagged BRAF-LgBiT or -SmBiT from HEK293T cells, both in the absence and presence of KRAS^G12V^. As expected from previous studies [29, 83], KRAS^G12V^ induced the typical phosphorylation mediated electrophoretic mobility shifts on KSR1 and BRAF with LgBiT or SmBiT extensions.

Next, we expressed the KSR1 fusion proteins in HEK293T cells and in combination with BRAF and RAF1 Nluc fusion proteins. These analyses provided several novel insights (Fig. 5C). First, KSR1-LgBiT and KSR1-SmBiT form homo-dimers in living cells, which supports previous co-immunoprecipitation experiments showing that the isolated N-terminal regulatory regions (NTRs) of the pseudokinase interact with each other [14]. Second and in contrast to BRAF homo- and heterodimers (Figs. 4A and 5B), KSR1-LgBiT/KSR1-SmBiT dimer formation was not promoted by KRAS^G12V^. This finding can be explained by the lack of an RBD in the pseudokinase (Fig. 5A) and fits to very recent insights showing that KSR1 membrane recruitment occurs independent of canonical RAS [17] and via the coiled coil and sterile alpha motif (CC-SAM) [51]. Nevertheless, it should be mentioned that the structurally distinct and more distant related RAS superfamily member DiRAS3 has been implicated in KSR1 homo-dimerization [84]. Interestingly, Sorafenib failed to augment KSR1 homodimerization (Fig. 5C), suggesting that this ATP competitive multi-kinase inhibitor neither binds to KSR1 nor induces dimer-promoting conformational changes in the pseudokinase domain, which is still able to accommodate ATP [17]. Moreover, KSR1, both in the LgBiT and SmBiT context, formed BRAF/KSR1 heterodimers, even in the absence of KRAS^G12V^ and/or Sorafenib (Fig. 5C). This observation is in line with Fig. 5B demonstrating already prominent levels of BRAF/KSR1 heterodimers in the absence of KRAS^G12V^. However, we were surprised that BRAF/KSR1 heterodimerization was not increased by Sorafenib in this setting (Fig. 5C), as others and we previously demonstrated that this compound promoted the interaction between ectopically expressed KSR1 and endogenous BRAF in co-immunoprecipitation experiments [29, 52]. Therefore, this discrepancy could be explained by the fact that we co-overexpressed BRAF and KSR1 in our NanoBiT assays, as we had to introduce the LgBiT/SmBiT moieties into both binding partners. On the other hand, it needs to be kept in mind that the NanoBiT approach reports PPIs in living cells, while the aforementioned co-immunoprecipitation experiments only reveal dimers stable enough to survive the purification procedure [29, 52]. In that regard, it is tempting to speculate that Sorafenib converts low-affinity BRAF/KSR1 dimers, which are readily detectable by the Nluc approach, into stable ones.

Interestingly, we observed little formation of RAF1/KSR1 heterodimers, both under basal conditions and in the presence of KRAS^G12V^ and/or Sorafenib. This could be explained by the finding that the NTRs of RAF1 and KSR1 barely interact [14]. As expected from previous studies using KSR1 without Nluc fusions [14, 28, 85], KSR1 augmented the MEK/ERK phosphorylation potential of BRAF-LgBiT and -SmBiT (Fig. 5D).

### The KSR1-BRAF salt bridge represents the major mediator of heterodimerization

**Fig. 6:**
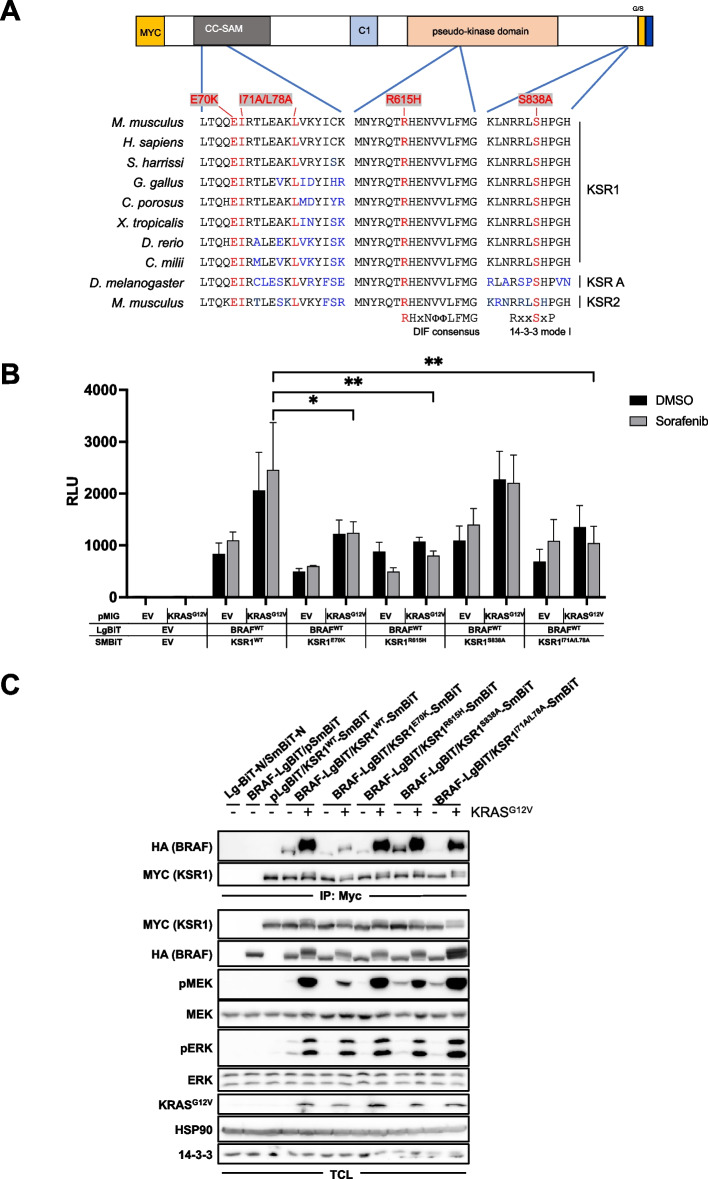
E70 of the CC-SAM domain of KSR1 plays a key role in its heterodimerization with BRAF. **A** Scheme of N-terminally Myc-tagged KSR1-SmBiT illustrating the localization of evolutionary conserved KSR1 PPI motifs tested in this study. The left alignment block shows the αC3-helix of the CC-SAM domain containing E70 forming a salt-bridge with BRAF [14], and I71 and L78 involved in membrane recruitment [51]. The middle block shows the highly conserved DIF core with the R615H mutation. The right block portrays the C-terminal mode I 14–3-3 binding motif. Mutated residues are shown in red and the individual amino acid substitutions are indicated. Residues differing from the reference (mKSR1) are highlighted in blue. **B** HEK293T cells were transfected with the indicated plasmid combinations and treated with 10 μM Sorafenib or DMSO for 4 h. Shown is the mean of four to seven biological replicates. **C** Expression vectors for the indicated Myc-tagged KSR1-SmBiT proteins and HA-tagged BRAF^WT^-LgBiT were co-transfected into HEK293T cells with either empty pMIG (-) or pMIG/KRAS^G12V^ ( +). KSR1-SmBiT fusion proteins were immunoprecipitated using anti-Myc antibody. Cells transfected with empty vectors or either pBRAF^WT^-LgBiT or pKSR^WT^-SmBiT serve as controls. Following Western blotting, IPs and TCLs were probed with the indicated antibodies to detect the specific interaction between KSR and BRAF and to confirm successful expression and pathway induction by KRAS^G12V^. Shown is a single experiment with this plasmid combination. The effect of the E70K mutation, however, was also observed in a similar experimental set-up involving the BRAF-SmBiT/KSR1-LgBiT combination

Next, we applied the split Nluc system to define the structural requirements underlying the prominent interaction between BRAF and KSR1. Therefore, we generated a series of expression vectors encoding KSR1-SmBiT fusion proteins in which residues previously implicated in the BRAF/KSR1 interaction or the equivalents of DIF and C-terminal 14–3-3 binding motif in RAF kinases were mutated. As there are five and three alternative transcripts reported for human and murine KSR1 in the ENSEMBL database, respectively, we illustrate the positions of the residues and motifs tested in Fig. 6A. Here, we refer to the numbering most frequently applied to murine KSR1, as its corresponding cDNA has been used in most studies [17, 51–54], incl. the present manuscript, for reasons outlined in Materials and Methods. Starting the description with the N-terminus of the protein, we generated the following mutants.

First, we introduced the E70K substitution into the CC-SAM domain of KSR1 (Fig. 6A), as Lavoie et al. demonstrated that E70 (reported as E72 in their publication) builds a salt-bridge with K88 located in the N-terminal BRAF specific region (BSR) [14]. By substituting E70 with a positively charged lysine residue, this salt-bridge with K88 can no longer be established. Using alanine substitutions, Koveal et al (2012) identified the adjacent residue, I71, together with L78, as critical residues for the membrane recruitment of KSR1 that could in turn contribute to the heterodimerization with membrane recruited BRAF [51]. To evaluate the relevance of the DIF of KSR1 for its heterodimerization with BRAF, we introduced the R615H substitution into its pseudokinase domain [52, 54]. R615H corresponds to R732H in Drosophila KSR, which led to the discovery of the critical and evolutionary conserved role of this arginine in RAF and KSR dimerization [28]. R615 represents a key residue of the aforementioned RHxNΦΦLFMG motif (Fig. 6A). This motif is found in the C-terminal half of the central DIF and is not only highly conserved among KSR ortho- and paralogues, but, as outlined above, also shared with RAF proteins [29]. Finally, to test the relevance of the equivalent of the C-terminal 14–3-3 binding site in the RAFs, we mutated S838 in KSR1 to alanine. S838 corresponds to S729 (BRAF) and S621 (RAF1) and its phosphorylation by PKA [86] creates a mode I 14–3-3 binding site [87].

We expressed these constructs in HEK293T cells, either singly or in combination with KRAS^G12V^, to study the KSR1/BRAF association by Nluc reconstitution (Fig. 6B) or in co- immunoprecipitation assays (Fig. 6C). To the best of our knowledge, this represents the first data set comparing all these KSR1 mutations side-by-side. We also noticed a high degree of BRAF/KSR1 heterodimerization with a trend for a further increase upon KRAS^G12V^ expression. Interestingly, this PPI was hardly influenced by the S838A mutation under all three conditions (basal, KRAS^G12V^, Sorafenib) and their combinations. This finding is in contrast to previous work showing that the S838A mutation reduces the co-immunoprecipitation efficacy between exogenous KSR1 and endogenous human BRAF in HEK293 cells [54]. Apart from the fact that we co-express both proteins in our setting and measure their interaction with the Nluc assay in living cells, this discrepancy might be explained by the fact that we applied milder lysis conditions (0.5% NP40) in the co-immunoprecipitation assay than Takahashi et al.(2017), who used 1% Triton X-100 [54]. In contrast, the E70K, the I71A/L78A double mutation and the R615H substitution showed a trend for reduced KSR1/BRAF association in the Nanoluc assays that became significant in the context of KRAS^G12V^ and Sorafenib treatment (Fig. 6B). Interestingly, the E70K mutation most prominently affected the BRAF/KSR1 association in the co-immunoprecipitation assay, especially upon co-expression with KRAS^G12V^ (Fig. 6C). This finding could be explained by a scenario in which BRAF is recruited to the membrane and converted into a dimerization competent state by KRASG12V, which in turn might facilitate salt-bridge formation between its BSR and the CC-SAM domain of KSR1.

The impact of the E70K mutation on the BRAF/KSR1 interaction in both Nluc and co-immunoprecipitation assays further indicates that the strong association between BRAF and KSR1 observed in our assays is specifically mediated by a defined PPI and does not represent an unspecific aggregation event potentially caused by overexpression. Moreover, the E70K, I71A/L78A and the R615H mutations significantly reduce the BRAF/KSR1 interaction in Sorafenib treated cells (Fig. 6B), suggesting that the aforementioned and prominent association of both proteins reflects a specific PPI and not simply an overexpression artifact.

In summary, our side-by-side comparison of these four KSR1 mutants suggest that the E70-K88 salt-bridge represents a critical stabilizer of BRAF/KSR1 heterodimers and that the DIF of the pseudokinase as well as its CC-SAM domain residues mediating membrane recruitment contribute to this PPI. Interestingly, in this and similar experiments, the KSR1 mutations had no discernible impact on ERK phosphorylation in KRAS^G12V^ expressing cells, suggesting that their impact on downstream signaling, which represents an interesting subject for future studies, could be overshadowed by this strong oncoprotein. In summary, our data demonstrate that C-terminal tagging of KSR1 with either LgBiT or SmBiT represents a feasible method to study its homo- and heterodimerization. This approach will be useful for the further definition of its role in various settings of ERK pathway regulation.

## Discussion

Protein–protein interactions (PPI) have been studied using biochemical approaches, such as co-immunoprecipitation assays, Far Western blot and Surface Plasmon Resonance Spectroscopy analyses or with genetic approaches such as (yeast) two hybrid systems. More recently, proximity ligation assays (PLA) were added to the toolkit allowing the detection of endogenous PPIs in situ [88]. While these methods continue to be extremely useful, they rely either on purified proteins, are based on cell-free systems or report, as in the case of two hybrid systems, static endpoints and are hence unsuitable to monitor the temporal dynamics of PPI. Likewise, time course series with PLA or co-immunoprecipitation experiments can provide an approximation of temporal dynamics, but are cumbersome for the tight monitoring during time course or dose titration experiments. Consequently, there is a strong interest in methods reporting PPI in real time and in living cells and approaches such as bimolecular fluorescence complementation [89] or split luciferase assays have received a lot of attention recently. Traditionally, RAF dimerization has been studied using co-immunoprecipitation experiments [21, 29, 42, 43] and PLA [41, 42]. While PLA relies on fixed and permeabilized cells and can only provide snapshots [88], co-immunoprecipitation analyses report only PPIs that are strong enough to survive lysis and numerous washing steps [90], which are required to discern specific interactors from contaminants. In contrast, the balanced on–off rate of split luciferase systems provides insights into weak and transient interactions, but of course their high sensitivity also raises the question whether their signal-to-noise ratio is high enough to deliver specific information. Consequently, we devised a series of experiments to evaluate the Nluc system for its ability to faithfully report differences in RAF homo- and hetero-dimerization under various conditions.

**Fig. 7:**
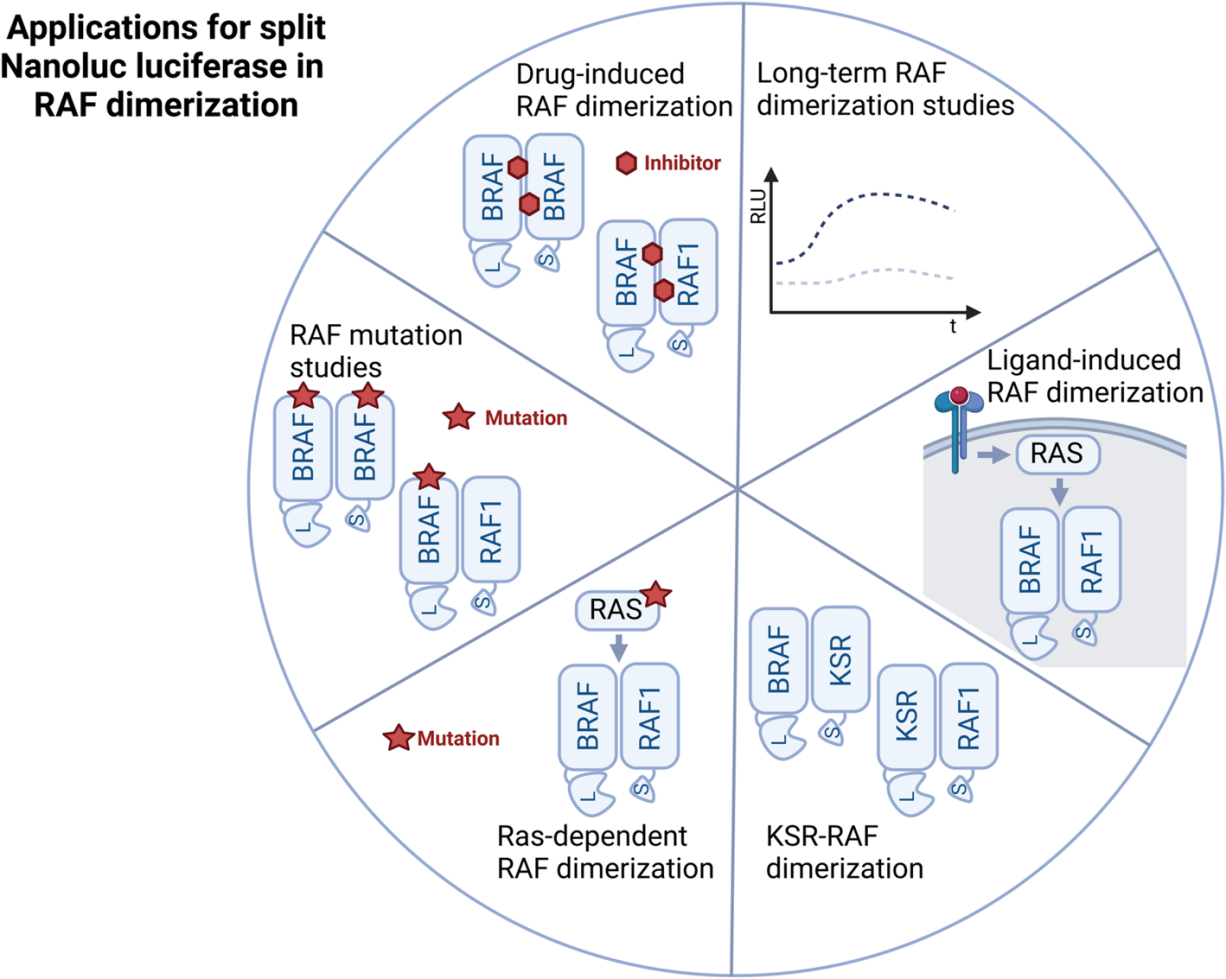
Cartoon depicting the different applications of the Nluc technology to study RAF dimerisation. See main text for details. Cartoon was generated using BioRender.com

In this study, we show that Nluc activity is reconstituted by well-known inducers of RAF dimerization such as EGF or oncogenic KRAS^G12V^ (Fig. 7). The specificity and biological meaning of these data is underscored by our experiments showing that well-established loss-of-function mutations in the RBD, the DIF and the C-terminal 14–3-3 binding motif all impair Nluc reconstitution. In contrast, the BRAF^V600E^ gain-of-function mutation that was shown to increase homo-dimerization, at least under post-lysis conditions [29, 36], displayed significantly increased Nluc reconstitution, which could be specifically reverted by the dimer disrupting paradox breaker PLX8394. In respect to RAFi, we confirm previous data by Dixon et al., who showed that GDC-0879 (type I) and Sorafenib (type II) effectively induce BRAF-LgBiT/RAF1-SmBiT heterodimers, while the Vemurafenib tool compound PLX4720 promoted weak Nluc activity only at high concentrations [48]. This suggested that the Nluc system is able to discern the properties of the three distinct types of RAFi. Here, we confirm and extend this notion by including additional and more recent RAFi of which several are currently in clinical trials or have already entered clinical practice. We show that type I^1/2^ compounds, incl. the recently approved Encorafenib, hardly induce BRAF/RAF1 heterodimers, while the third generation and type II RAFi Belvarafenib, Lifirafenib, MLN2480, TAK-632 and Naporafenib significantly promoted BRAF/RAF1 heterodimers. In summary, we provide several genetic and pharmacological proof-of-concepts that the Nluc system is suitable to monitor different degrees of RAF homo- and hetero-dimerization and might be useful to decipher the mode of action of newly identified RAFi. Using the more stable Nluc substrates Vivazine and Endurazine, we show that BRAF-LgBiT/RAF1-SmBiT pairs stabilized by the potent dimer inducing type II inhibitor Naporafenib maintain their activity for at least 24 h. This proof-of-concept for a long-term observation and the observation by Dixon that RAFi washout rapidly reverses Nluc activity [48] demonstrate that this 96-well based dimerization reporter system can be used for extended time course experiments. Such data can then inform about drug and/or target protein stability, the best harvesting time point or the appropriate drug concentration to conduct large scale proteomic experiments. It is also tempting to speculate that the split Nluc system could be used in vivo, e.g. to monitor drug induced dimerization in xenografts in correlation with pharmacokinetic and -dynamic analyses. Although addressing this concept represents a study in its own right, the recently reported in vivo applications of Nluc split luciferase system to detect the binding of SmBiT containing oncolytic viruses to LgBiT expressing tumor cells support this concept [91].

Despite these potentials, our extensive analysis identified some limitations that are worth considering to improve future applications of the split Nluc luciferase system. As it is commonly observed with highly sensitive luciferase assays, there is often a considerable degree of variation between biological replicates and it is not uncommon that averages of technical replicates are represented from a single representative experiment. As reflected by our statistical analyses, we noticed a certain variability between the fold changes of our biological replicates, which were in some cases conducted over several months apart from each other. As pointed out in the Results section, however, significant effects were observed for many comparisons and, if no significance was detected across biological replicates, most mutations and drug treatments still provoked clear and reproducible trends that fit to mechanisms proposed by other experimental approaches. Variability in luciferase assays can result from various factors such as differences in substrate batches or (co-)transfection efficiency, in particular if several plasmids are involved as it is also the case in our experimental set-ups. The latter source of variation could be minimized by using constructs in which expression of LgBiT and SmBiT is driven from a bidirectional promoter, as it was reported more recently by the manufacturer of Nluc expression plasmids on their website. Once the most important LgBiT/SmBiT combination is identified, stably transfected cell lines might represent another improvement, in particular if only one or few reporter lines are required and comparable expression of the reporter proteins is maintained over extended periods of time. Ultimately, CRISPR/Cas mediated knock-ins of the LgBiT and SmBiT cassettes into endogenous gene loci might be an elegant approach for selected interaction pairs. Nevertheless, such an approach is not feasible for larger interaction screens in which, for example, residues in interaction interfaces shall be functionally validated.

A second important point to consider in designing split Nluc reporter assays is the positioning of the Nluc moieties and the use of linker segments, usually a G/S-rich stretch. Based on previous experiences by other groups designing and using various split luciferase systems in the context of RAF kinases [44, 48] and based on recent cryo-EM data revealing the quaternary structure of RAF complexes [22], we did not fuse LgBiT and SmBiT to the N-termini of RAF proteins, as we did not expect enough proximity between both Nluc moieties in this set-up. Dixon et al. have used the BRAF-LgBiT/RAF1-SmBiT combination for their initial demonstration of the split Nluc system [48] and while our manuscript was in an advanced stage of preparation, Murphy et al. also applied this pair to demonstrate the potent induction of BRAF/RAF1 heterodimers in MEFs harboring oncogenic *Nras* knock-in alleles [73]. We confirm the efficient BRAF-LgBiT/RAF1-SmBiT heterodimerization for other experimental conditions such as EGF stimulation, expression of oncogenic KRAS^G12V^, various RAF1 mutants and long-term drug exposure experiments. Interestingly, however, we observed in the course of this project that the fold-changes in BRAF/RAF1 heterodimerization will be further enhanced, if BRAF is fused to SmBiT instead. Likewise, we noticed that the signaling output of BRAF^WT^ or BRAF^V600E^ was higher in the SmBiT than the LgBiT context. The underlying mechanism for these discernible but rather subtle effect remain unclear. Nevertheless, as cells expressing BRAF-LgBiT, BRAF-SmBiT, RAF1-LgBiT and RAF1-SmBiT, either singly or in combination, still display elevated MEK/ERK phosphorylation levels compared to empty vector control cells, we can rule out major signaling deficits for these proteins, incl. BRAF-LgBiT. This observation, however, highlights the need to consider the possibility that, compared to GFP or other split luciferases, even relatively small fusion partners such as LgBiT might affect the interactome and/or stability of the protein under investigation. Therefore, the design and use of reporter constructs should be precisely described and Nluc experiments should be accompanied by Western blot analyses confirming the degree of expression and functionality of the fusion proteins. Lastly, PPIs reported by the split Nluc system should not be considered in isolation for the construction of working hypotheses, as the high sensitivity of the split Nluc system is able to detect PPIs of lower affinity that are potentially irrelevant for downstream signaling, Thus, not every RAF dimer detected in split Nluc assays is necessarily functional, as it is demonstrated by the low-level association of the signaling impaired DIF and 14–3-3 binding mutants (Fig. 2).

In summary, we have demonstrated that the split Nluc system is suitable to dissect the structural requirements underlying the homo- and heterodimerization of the BRAF and RAF1 isoforms and their interaction with KSR1. The system allows the evaluation of gain- and loss-of-function mutations of RAF and KSR proteins as well as the influence of RAFi on dimerization (Fig. 7). Together with reporters monitoring RAS/RAF interaction [72, 92], intramolecular conformational changes of RAF kinases [93, 94] and (in)direct ERK activation reporters [66, 95–97], there is now an impressive toolkit for *in cellulo* analysis of RAS/ERK pathway dynamics available that will aid in addressing various physiological, pathological and pharmacological aspects of this important signaling axis.

## Supplementary Information

**Additional file 1: Figure S1.** Co-immunoprecipitation using anti-c-Myc antibodies confirms the enhanced heterodimerization between BRAF-LgBiT and RAF1-SmBiT as well as between RAF1-LgBiT and BRAF-SmBiT proteins in the presence of either KRAS^G12V^ or Sorafenib. HEK293T cells were co-transfected with the indicated plasmids and either pMIG empty vectoror pMIG/KRAS^G12V^. Four hours prior to lysis, cells were treated with either 10 μM Sorafenib or the equivalent volume of DMSO. Myc-tagged RAF1-SmBiT or RAF1-LgBiT proteins were immuno-purified using anti-Myc antibody. Top: Immunoprecipitates were loaded on two gels. Purified RAF1 proteins were either detected using anti-Myc or RAF1 antibodies, while co-purified HA-tagged BRAF proteins were detected with anti-HA or anti-BRAF F7 antibodies. Note the expected increased BRAF/RAF1 heterodimerization by KRAS^G12V^ or sorafenib. Bottom: Analysis of total cellular lysates demonstrates expression of all Nluc components and confirms activation of the RAS/RAF/MEK/ERK-pathway by KRAS^G12V^.

**Additional file 2: Figure S2.** Side-by-side comparison of growth factor versus oncogenic KRAS^G12V^ induced BRAF-LgBiT/RAF1-SmBIT heterodimerization. HEK293T cells were transfected with either empty pMIGor pMIG/KRAS^G12V^ and the empty control vectors pLgBiT-N/pSmBit-N or the BRAF-LgBiT and RAF1-SmBiT expression vectors. Cells were either left untreated or stimulated with 100 ng/ml EGF for the indicated timepoints. Note the EGF induced increase in cells expressing BRAF-LgBiT/RAF1-SmBiT that is absent in cells transfected with the empty control vectors. Also note the consistent and highly significant increase in Nluc activity in unstimulated KRAS^G12V^ expressing cells compared to EGF stimulated cells transfected with pMIG e.V.

**Additional file 3: Figure S3.** Effects of more complex DIF mutations in BRAF on its heterodimerization with RAF1. HEK293T cells were transfected with either empty pLgBiT-N/pSmBiT-N control plasmidsor expression vectors encoding RAF1^WT^-SmBiT and the indicated BRAF-LgBiT proteins, either in combination with pMIG e.V.or pMIG KRAS^G12V^. Cells were either treated with 10 µM Sorafenib or DMSO for 4 h prior to measurement. Shown is the mean of three to four biological replicates. HEK293T cells were transfected with either empty pLgBiT-N/pSmBiT-N control plasmids or expression vectors encoding RAF1^WT^-SmBiT and the indicated BRAF-LgBiT proteins, either in combination with pMIG e.V. or pMIG KRAS^G12V^. Cells were either treated with 10 µM Sorafenib or DMSO) for 4 h prior to measurement. RAF1^WT^-SmBiT fusion proteins were immunoprecipitated using anti-Myc antibody. Following Western blotting, immunoprecipitatesand TCLs were probed with anti-HA and anti-Myc antibodies. Shown is a representative experiment from two biological replicates.

**Additional file 4.**

## Data Availability

This study does not associate with large datasets such as proteomic or transcriptomic data that would be deposited on servers. Data or material are available from the corresponding author upon reasonable request.
