# Supplementary figures and images for: Analysis of RAS and drug induced homo- and heterodimerization of RAF and KSR1 proteins in living cells using split Nanoluc luciferase
Source: Cell Commun Signal. 2023 Jun 14;21:136. doi: 10.1186/s12964-023-01146-9 (PMC10265822; doi:10.1186/s12964-023-01146-9)

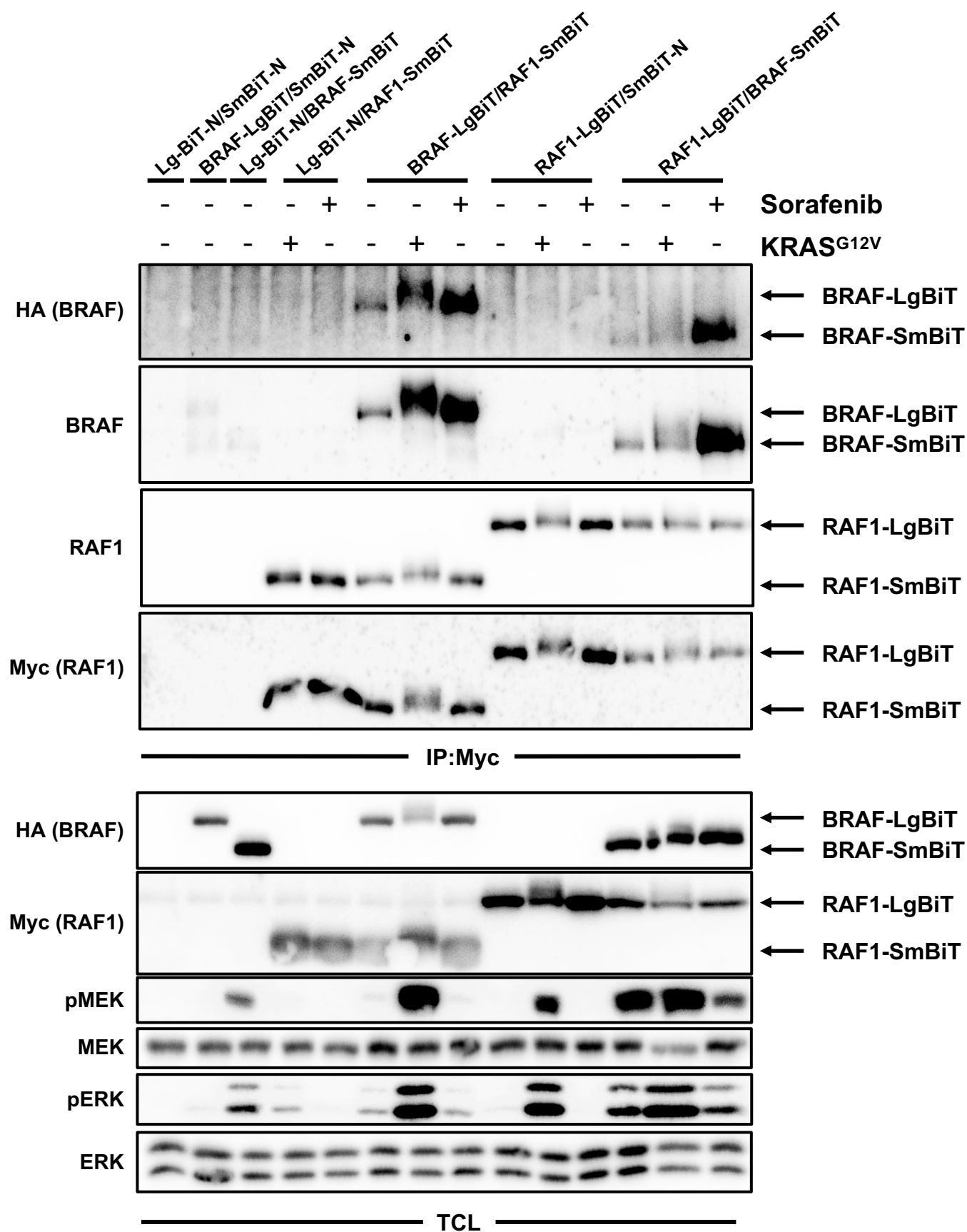

Supplement: Supplementary file 2 — Additional file 1: Figure S1. Co-immunoprecipitation using anti-c-Myc antibodies confirms the enhanced heterodimerization between BRAF-LgBiT and RAF1-SmBiT as well as between RAF1-LgBiT and BRAF-SmBiT proteins in the presence of either KRASG12V or Sorafenib. HEK293T cells were co-transfected with the indicated plasmids and either pMIG empty vectoror pMIG/KRASG12V. Four hours prior to lysis, cells were treated with either 10 μM Sorafenib or the equivalent volume of DMSO. Myc-tagged RAF1-SmBiT or RAF1-LgBiT proteins were immuno-purified using anti-Myc antibody. Top: Immunoprecipitates were loaded on two gels. Purified RAF1 proteins were either detected using anti-Myc or RAF1 antibodies, while co-purified HA-tagged BRAF proteins were detected with anti-HA or anti-BRAF F7 antibodies. Note the expected increased BRAF/RAF1 heterodimerization by KRASG12V or sorafenib. Bottom: Analysis of total cellular lysates demonstrates expression of all Nluc components and confirms activation of the RAS/RAF/MEK/ERK-pathway by KRASG12V. [file 12964_2023_1146_MOESM1_ESM.pdf]

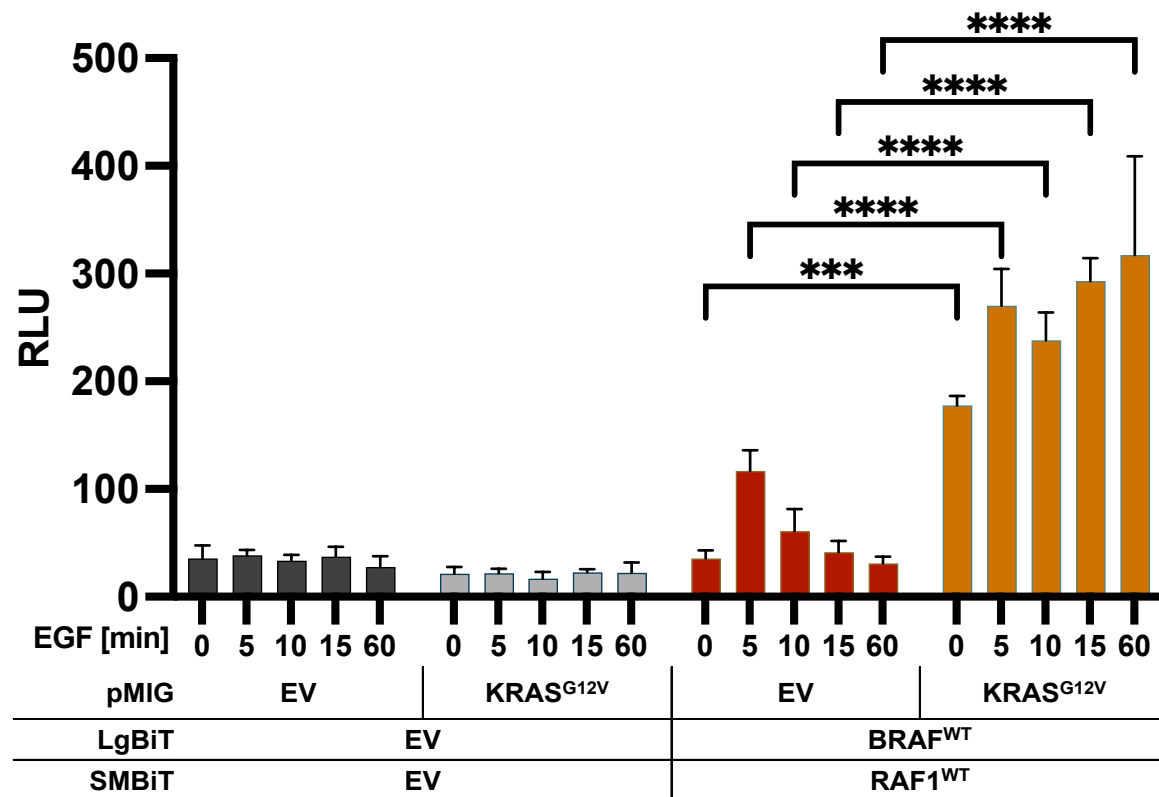

Supplement: Supplementary file 3 — Additional file 2: Figure S2. Side-by-side comparison of growth factor versus oncogenic KRASG12V induced BRAF-LgBiT/RAF1-SmBIT heterodimerization. HEK293T cells were transfected with either empty pMIGor pMIG/KRASG12V and the empty control vectors pLgBiT-N/pSmBit-N or the BRAF-LgBiT and RAF1-SmBiT expression vectors. Cells were either left untreated or stimulated with 100 ng/ml EGF for the indicated timepoints. Note the EGF induced increase in cells expressing BRAF-LgBiT/RAF1-SmBiT that is absent in cells transfected with the empty control vectors. Also note the consistent and highly significant increase in Nluc activity in unstimulated KRASG12V expressing cells compared to EGF stimulated cells transfected with pMIG e.V. [file 12964_2023_1146_MOESM2_ESM.pdf]

**A**

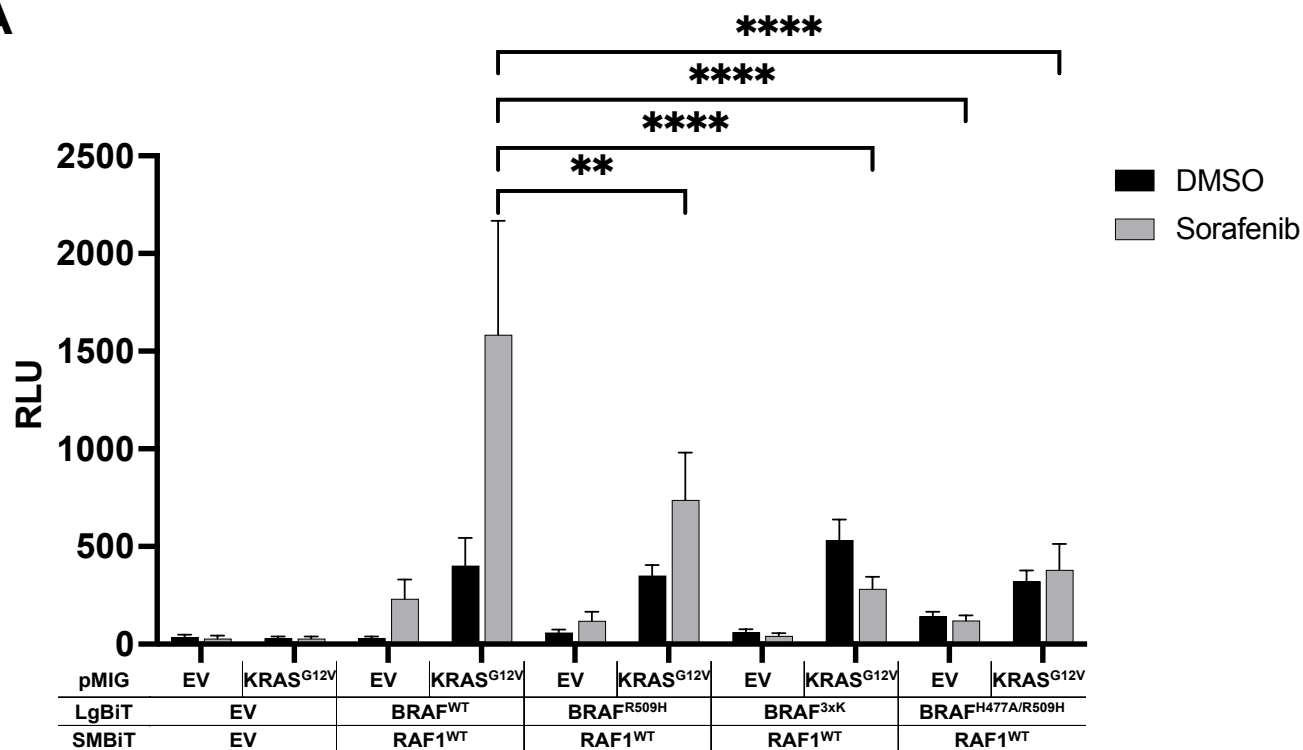

**B**

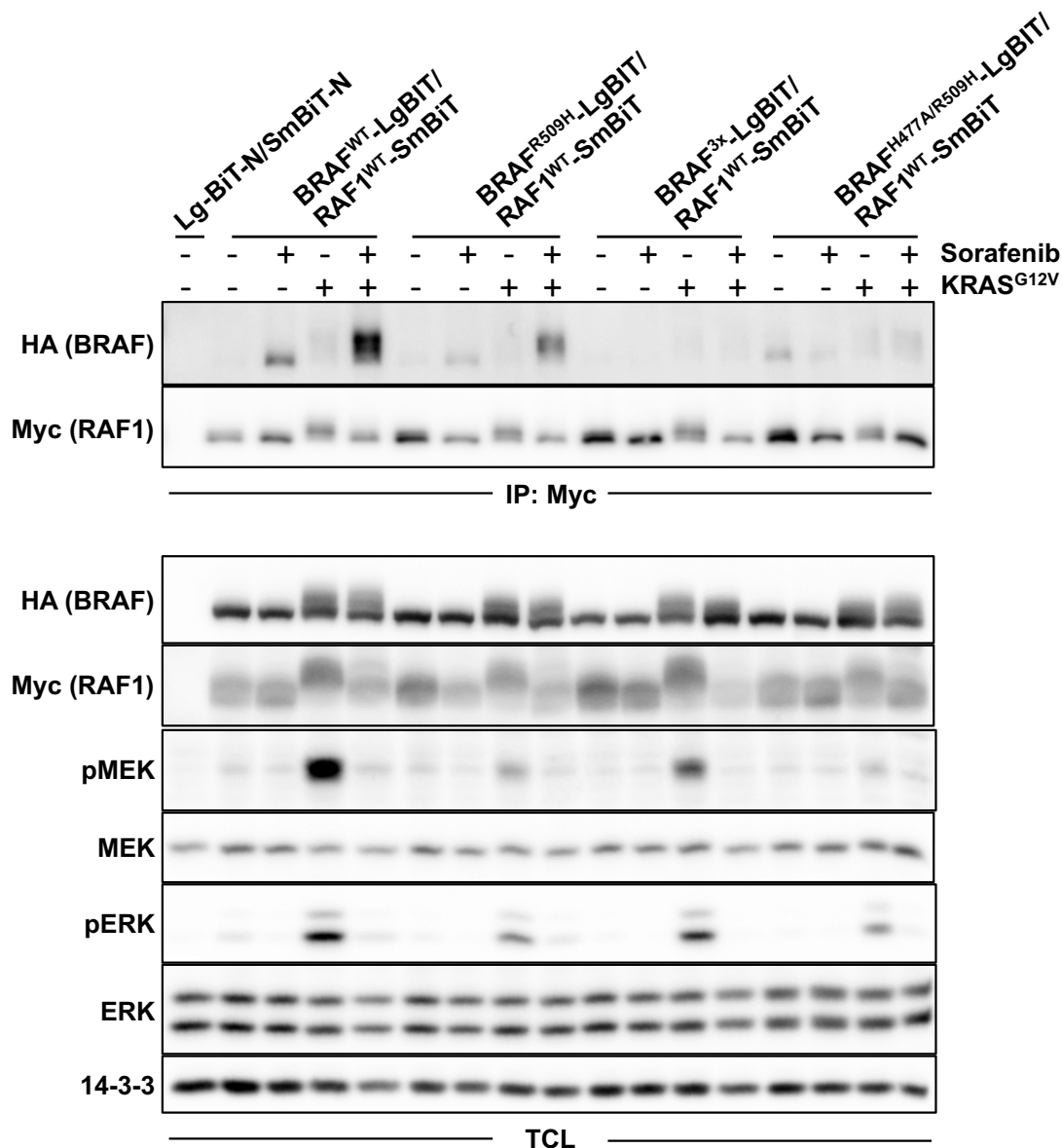

Supplement: Supplementary file 4 — Additional file 3: Figure S3. Effects of more complex DIF mutations in BRAF on its heterodimerization with RAF1. HEK293T cells were transfected with either empty pLgBiT-N/pSmBiT-N control plasmidsor expression vectors encoding RAF1WT-SmBiT and the indicated BRAF-LgBiT proteins, either in combination with pMIG e.V.or pMIG KRASG12V. Cells were either treated with 10 µM Sorafenib or DMSO for 4 h prior to measurement. Shown is the mean of three to four biological replicates. HEK293T cells were transfected with either empty pLgBiT-N/pSmBiT-N control plasmids or expression vectors encoding RAF1WT-SmBiT and the indicated BRAF-LgBiT proteins, either in combination with pMIG e.V. or pMIG KRASG12V. Cells were either treated with 10 µM Sorafenib or DMSO) for 4 h prior to measurement. RAF1WT-SmBiT fusion proteins were immunoprecipitated using anti-Myc antibody. Following Western blotting, immunoprecipitatesand TCLs were probed with anti-HA and anti-Myc antibodies. Shown is a representative experiment from two biological replicates. [file 12964_2023_1146_MOESM3_ESM.pdf]
