# Supplementary material for: Analysis of RAS and drug induced homo- and heterodimerization of RAF and KSR1 proteins in living cells using split Nanoluc luciferase
Source: Cell Commun Signal. 2023 Jun 14;21:136. doi: 10.1186/s12964-023-01146-9 (PMC10265822; doi:10.1186/s12964-023-01146-9)
Supplement: Supplementary file 5 — Additional file 4. [file 12964_2023_1146_MOESM4_ESM.pdf]

Table S1. Sequences of oligonucleotides used for mutagenesis in 5'➔3' direction

BRAF R188L fwd	GACAGTCTAAAGAAAGCACTGATGATGCTAGGTCTAATCCCAGAGTGCTG
BRAF R188L rev	CAGCACTCTGGGATTAGACCTAGCATCATCAGTGCTTTCTTTAGACTGTC
BRAF H477A fwd	CAGTCTACAAGGGAAAGTGGGCTGGTGATGTGGCAGTGAAAATG
BRAF H477A rev	CATTTTCACTGCCACATCACCAGCCCACTTTCCCTTGTAGACTG
BRAF R509H fwd	GAAGTAGGAGTACTCAGGAAAACACATCATGTGAATATCCTACTCTTCATGGG
BRAF R509H rev	CCCATGAAGAGTAGGATATTCACATGATGTGTTTTCCTGAGTACTCCTACTTC
BRAF 3x fwd	GGAAAACACATCATGTGAATATCCTAGGCTTCTGGGGCTATTCCACAAAGCCACAA CTGG
BRAF 3x rev	CCAGTTGTGGCTTTGTGGAATAGCCCCAGAAGCCTAGGATATTCACATGATGTGTT TTCC
BRAF S729A fwd	GCCAAAAATTCACCGCAGTGCAGCAGAACCCTCCTTGAATCGGGCTGG
BRAF S729A rev	CCAGCCCGATTCAAGGAGGGTTCTGCTGCACTGCGGTGAATTTTTGGC
mKSR1 E70K fwd	CTAACGACCTCACACAGCAGAAGATCCGGACCCTAGAGGCAAAG
mKSR1 E70K rev	CTTTGCCTCTAGGGTCCGGATCTTCTGCTGTGTGAGGTCGTTAG
mKSR1 R615H fwd	GTGATGAACTACCGGCAGACGCACCATGAGAACGTGGTGCTCTTCATG
mKSR1 R615H rev	CATGAAGAGCACCACGTTCTCATGGTGCGTCTGCCGGTAGTTCATCAC
mKSR1 S838A fwd	CCAAGCTGAACCGGCGGCTCGCCCACCCTGGGCACTTTTGG
mKSR1 S838A rev	CCAAAAGTGCCCAGGGTGGGCGAGCCGCCGGTTCAGCTTGG
mKSR1 I71AL78A fwd	CTAACGACCTCACACAGCAGGAGGCCCGGACCCTAGAGGCAAAGGCGGTGAAAT ACATTTGCAAGCAG
mKSR1 I71AL78A rev	CTGCTTGCAAATGTATTTCACCGCCTTTGCCTCTAGGGTCCGGGCCTCCTGCTGTG TGAGGTCGTTAG
RAF1 R89L fwd	CTTATGAAAGCACTCAAGGTGCTGGGCCTGCAACCAGAGTG
RAF1 R89L rev	CACTCTGGTTGCAGGCCCAGCACCTTGAGTGCTTTCATAAG
RAF1 H369A fwd	GAACTGTTTATAAGGGTAAATGGGCCGGAGATGTTGCAGTAAAG
RAF1 H369A rev	CTTTACTGCAACATCTCCGGCCCATTTACCCTTATAAACAGTTC
RAF1 R401H fwd	GAGGTGGCTGTTCTGCGCAAAACACATCATGTGAACATTCTGCTTTTCATGGGG
RAF1 R401H rev	CCCCATGAAAAGCAGAATGTTCACATGATGTGTTTTGCGCAGAACAGCCACCTC
RAF1 S621A fwd	CGAAGATCAACCGGAGCGCTGCCGAGCCATCCTTGCATCG
RAF1 S621A rev	CGATGCAAGGATGGCTCGGCAGCGCTCCGGTTGATCTTCG
